# Catalytic Cycle of the Bifunctional Enzyme Phosphoribosyl-ATP Pyrophosphohydrolase/Phosphoribosyl-AMP Cyclohydrolase

**DOI:** 10.1021/acscatal.3c01111

**Published:** 2023-05-23

**Authors:** Gemma Fisher, Ennio Pečaver, Benjamin J. Read, Susannah K. Leese, Erin Laing, Alison L. Dickson, Clarissa M. Czekster, Rafael G. da Silva

**Affiliations:** School of Biology, University of St Andrews, Biomedical Sciences Research Complex, St Andrews, Fife KY16 9ST, U.K.

**Keywords:** phosphoribosyl-ATP pyrophosphohydrolase, phosphoribosyl-AMP cyclohydrolase, *Acinetobacter baumannii*, enzyme kinetics, histidine biosynthesis, kinetic isotope effects, substrate channeling

## Abstract

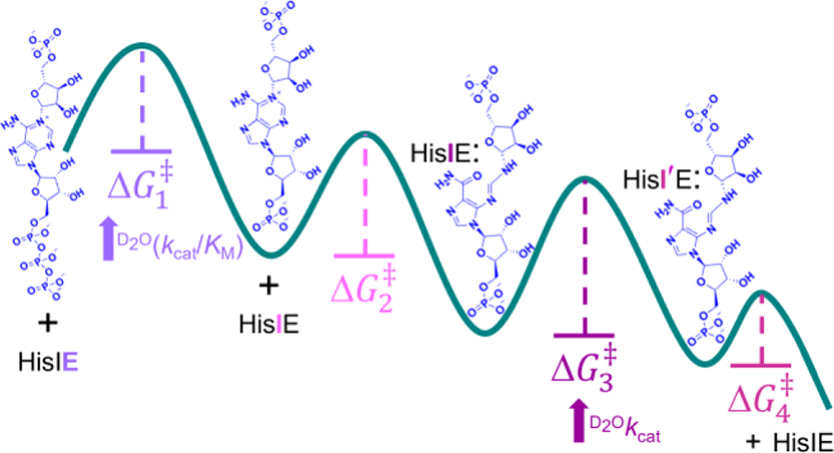

The bifunctional enzyme phosphoribosyl-ATP pyrophosphohydrolase/phosphoribosyl-AMP cyclohydrolase (HisIE) catalyzes the second and third steps of histidine biosynthesis: pyrophosphohydrolysis of *N*^1^-(5-phospho-β-D-ribosyl)-ATP (PRATP) to *N*^1^-(5-phospho-β-D-ribosyl)-AMP (PRAMP) and pyrophosphate in the C-terminal HisE-like domain, and cyclohydrolysis of PRAMP to *N*-(5′-phospho-D-ribosylformimino)-5-amino-1-(5″-phospho-D-ribosyl)-4-imidazolecarboxamide (ProFAR) in the N-terminal HisI-like domain. Here we use UV–VIS spectroscopy and LC–MS to show *Acinetobacter baumannii* putative HisIE produces ProFAR from PRATP. Employing an assay to detect pyrophosphate and another to detect ProFAR, we established the pyrophosphohydrolase reaction rate is higher than the overall reaction rate. We produced a truncated version of the enzyme-containing only the C-terminal (HisE) domain. This truncated HisIE was catalytically active, which allowed the synthesis of PRAMP, the substrate for the cyclohydrolysis reaction. PRAMP was kinetically competent for HisIE-catalyzed ProFAR production, demonstrating PRAMP can bind the HisI-like domain from bulk water, and suggesting that the cyclohydrolase reaction is rate-limiting for the overall bifunctional enzyme. The overall *k*_cat_ increased with increasing pH, while the solvent deuterium kinetic isotope effect decreased at more basic pH but was still large at pH 7.5. The lack of solvent viscosity effects on *k*_cat_ and *k*_cat_/*K*_M_ ruled out diffusional steps limiting the rates of substrate binding and product release. Rapid kinetics with excess PRATP demonstrated a lag time followed by a burst in ProFAR formation. These observations are consistent with a rate-limiting unimolecular step involving a proton transfer following adenine ring opening. We synthesized *N*^1^-(5-phospho-β-D-ribosyl)-ADP (PRADP), which could not be processed by HisIE. PRADP inhibited HisIE-catalyzed ProFAR formation from PRATP but not from PRAMP, suggesting that it binds to the phosphohydrolase active site while still permitting unobstructed access of PRAMP to the cyclohydrolase active site. The kinetics data are incompatible with a build-up of PRAMP in bulk solvent, indicating HisIE catalysis involves preferential channeling of PRAMP, albeit not via a protein tunnel.

## Introduction

**Scheme 1:**
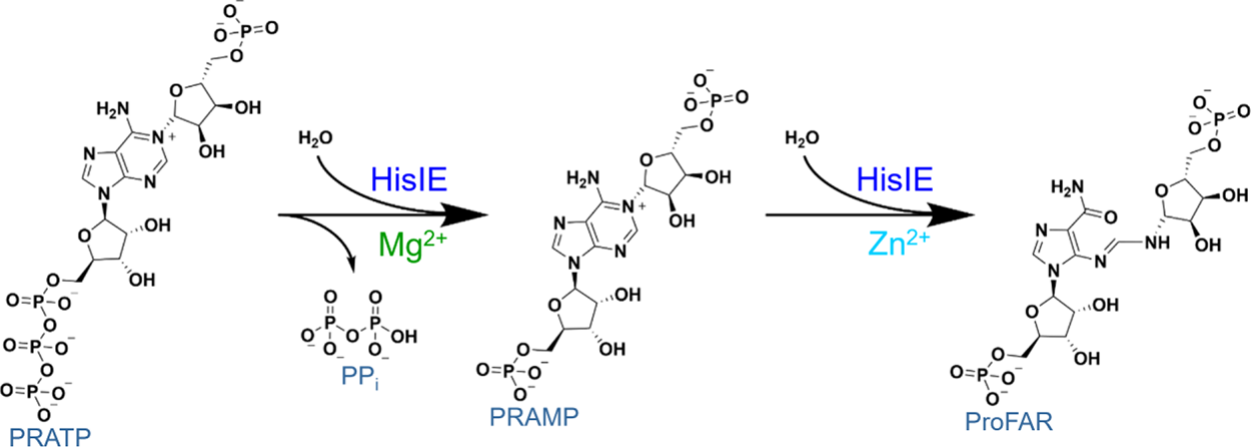
HisIE-catalyzed Hydrolysis of PRATP Followed by Ring-Opening Hydrolysis of PRAMP

The first step of histidine biosynthesis comprises the reversible condensation of ATP and 5-phospho-α-D-ribosyl-1-pyrophosphate (PRPP) to generate *N*^1^-(5-phospho-β-D-ribosyl)-ATP (PRATP) and pyrophosphate (PP_i_), catalyzed by ATP phosphoribosyltransferase (ATPPRT).^1^ In some actinobacteria, such as *Mycobacterium tuberculosis* (*M. tuberculosis*), and in most archaea, the second and third steps of histidine biosynthesis are catalyzed by the enzymes phosphoribosyl-ATP pyrophosphohydrolase (HisE) and phosphoribosyl-AMP cyclohydrolase (HisI), respectively. However, in most bacteria, including *Acinetobacter baumannii* (*A. baumannii*),^2^ the two reactions are catalyzed by the bifunctional enzyme phosphoribosyl-ATP pyrophosphohydrolase/phosphoribosyl-AMP cyclohydrolase (HisIE), comprised of an N-terminal domain homologous to HisI and a C-terminal domain homologous to HisE.^3^ HisIE catalyzes the Mg^2+^-dependent hydrolysis of PRATP to *N*^1^-(5-phospho-β-D-ribosyl)-AMP (PRAMP) and PP_i_, presumably in the C-terminal (HisE-like) domain, and the Zn^2+^-dependent ring-opening hydrolysis of PRAMP to *N*-(5′-phospho-D-ribosylformimino)-5-amino-1-(5″-phospho-D-ribosyl)-4-imidazolecarboxamide (ProFAR), presumably in the N-terminal (HisI-like) domain (Scheme 1).^4^

Crystal structures of *M. tuberculosis* monofunctional HisE (PDB ID: 1Y6X)^5^ and *Methanococcus thermoautotrophicum* (*M. thermoautotrophicum*) monofunctional HisI (PDB ID: 1ZPS)^6^ have been reported, as were those of apoenzyme and AMP-bound bifunctional HisIE from *Shigella flexneri* (PDB ID: 6J22 and 6J2L)^7^ and HisN2 (the nomenclature adopted in plants) *Medicago truncatula* (PDB ID: 7BGM and 7BGN).^8^ Mechanistic investigations of HisI have been described,^9,10^ but no detailed functional characterization has been reported for HisE or HisIE.

Enzymes involved in the histidine biosynthesis pathway are attractive targets for antibiotic development, as they carry out essential functions during infection and have no homologues in humans. For instance, histidine biosynthesis protects *M. tuberculosis* from host-imposed starvation.^11^ In *A. baumannii*, high-throughput transposon library analysis demonstrated that six enzymes of the histidine biosynthetic pathway, including HisIE, are required for the bacterium’s persistence in the lungs during pneumonia.^2^ In an independent study, knockout of the gene encoding another enzyme in the pathway, imidazole glycerol phosphate synthase, significantly increased host survival in a murine model of pneumonia caused by *A. baumannii*.^12^ Histidine is required for zinc acquisition and lung infection by *A. baumannii*,^13^ and it is a precursor in the biosynthesis of acinetobactin, a siderophore essential for *A. baumannii* virulence.^14^ Extracellular histidine concentration is lower than 2 μM in the lungs of mice, regardless of *A. baumannii* infection,^13^ while the histidine inhibition constant for *A. baumannii* ATPPRT, the enzyme allosterically inhibited by the amino acid in a negative feedback control mechanism, lies between 83 and 282 μM.^15^ As this value is expected to reflect the metabolite concentration the cell needs to function,^16^ this may explain the reliance of this bacterium on histidine biosynthesis to establish and sustain pneumonia.^15^

The need for novel antibiotics effective against carbapenem-resistant *A. baumannii* was classified as a critical priority by the World Health Organisation.^17^ Moreover, ventilator-associated pneumonia is one of the most common manifestations of *A. baumannii* infection, and it is linked to high mortality rates.^18^ The identification and characterization of promising novel molecular targets are key steps to enable rational drug design.^19^ Thus, the characterization of *A. baumannii* HisIE (*Ab*HisIE) catalysis may lay the foundation for inhibitor design against this enzyme on the path toward novel antibiotics against *A. baumannii*.

Here, the gene encoding the putative *Ab*HisIE was cloned and expressed, and the recombinant protein was purified. Liquid chromatography-mass spectrometry (LC–MS), differential scanning fluorimetry (DSF), steady-state and pre-steady-state kinetics, biocatalytic syntheses of PRAMP and *N*^1^-(5-phospho-β-D-ribosyl)-ADP (PRADP), solvent deuterium kinetic isotope effects, and viscosity effects were used to characterize the enzyme and its catalyzed reaction. Furthermore, the HisE-like domain of *Ab*HisIE (heretofore referred to as *Ab*HisE^domain^) was cloned and shown to catalyze the reaction normally catalyzed by monofunctional HisE, paving the way to study the pyrophosphohydrolysis reaction independently.

## Materials and Methods

### Materials

All commercially available chemicals were used without further purification. BaseMuncher endonuclease was purchased from Abcam. Ampicillin, dithiothreitol (DTT), and isopropyl-β-D-1-thiogalactopyranoside (IPTG) were purchased from Formedium. *Escherichia coli* (*E. coli*) DH5α (high efficiency) and BL21(DE3) cells, Gibson Assembly Cloning Kit, and Dpn1 were purchased from New England Biolabs. QIAprep Spin Miniprep, PCR clean-up, and Plasmid Midi kits were purchased from Qiagen. Ethylenediaminetetraacetic acid (EDTA)-free Complete protease inhibitor cocktail was purchased from Roche. Ammonium bicarbonate, ammonium sulfate, ATP, deuterium oxide (D_2_O), glycerol, histidine, imidazole, lysozyme, PRPP, potassium chloride, d-ribose 5-phosphate, tricine, phosphoenolpyruvate, *Saccharomyces cerevisiae* (*S. cerevisiae*) pyruvate kinase (*Sc*PK), *S. cerevisiae* myokinase (*Sc*MK), NiCl_2_, and ZnCl_2_ were purchased from Merck. Agarose, dNTPSs, kanamycin, 4-(2-hydroxyethyl)piperazine-1-ethanesulfonic acid (HEPES) EnzCheck Pyrophosphate Kit, MgCl_2_, NaCl, PageRuler Plus Prestained protein ladder, and Phusion High-Fidelity polymerase were purchased from ThermoFisher Scientific. *Psychrobacter arcticus* HisG_S_ (*Pa*HisG_S_), *M. tuberculosis* pyrophosphatase (*Mt*PPase), and tobacco etch virus protease (TEVP) were produced as previously described,^20^ as was *E. coli* PRPP synthetase (*Ec*PRPPS).^21^

### Expression of *Ab*HisIE and *Ab*HisE^domain^

The DNA encoding *Ab*HisIE (*A. baumannii* strain ATCC17978) and *Ab*HisE^domain^, codon optimized for expression in *E. coli*, were purchased as gBlocks (IDT). The *Ab*HisE^domain^ gBlock also encoded a TEVP-cleavable N-terminal His-tag. Each gBlock was PCR-modified and inserted into a modified pJexpress414 plasmid using Gibson Assembly^22^ according to the manufacturer’s instructions. Each construct was transformed into DH5α competent cells, sequenced (DNA Sequencing & Services, University of Dundee) to confirm the insertion of the genes and that no mutations had been introduced. Constructs were transformed into *E. coli* BL21(DE3) cells, which were grown in lysogeny broth (LB) containing 100 μg mL^–1^ ampicillin at 37 °C until an optical density at 600 nm (OD_600_) of 0.6–0.8, at which point cells harboring the *Ab*HisIE expression construct were equilibrated to 20 °C, while cells harboring the *Ab*HisE^domain^ expression construct remained at 37 °C before expression was induced with 0.5 mM IPTG. Cells were grown for an additional 20 h, harvested by centrifugation (6774 *g*, 15 min, 4 °C) and stored at −20 °C. The genes encoding *Ab*HisIE and *Ab*HisE^domain^ were cloned and expressed independently of one another.

### Purification of *Ab*HisIE and *Ab*HisE^domain^

All purification procedures were performed on ice or at 4 °C using an ÄTKA Start FPLC system (GE Healthcare). All SDS-PAGE used a NuPAGE Bis-Tris 4–12% Precast Gel (ThermoFisher Scientific). For *Ab*HisIE purification, cells were resuspended in buffer A (50 mM HEPES pH 7.5) supplemented with 0.2 mg mL^–1^ lysozyme, 750 U BaseMuncher endonuclease, and half a tablet of EDTA-free Complete protease inhibitor cocktail. Cells were lysed in a cell disruptor (Constant systems) at 30 kpsi, and centrifuged at 48,000 *g* for 30 min to remove cell debris. *Ab*HisIE was precipitated by dropwise addition of 1.5 M ammonium sulfate in buffer A to the supernatant followed by stirring for 1 h. The sample was centrifuged at 48,000 *g* for 30 min, the supernatant was discarded, and the pellet was resuspended in buffer A, dialyzed against 3 × 2 L of buffer A, filtered through a 0.45 μm membrane and loaded onto a 10 mL HiTrap Q FF column pre-equilibrated with buffer A. The column was washed with 10 column volumes(CV) of 2.5% buffer B (50 mM HEPES pH 7.5, 2 M NaCl) and the adsorbed proteins were eluted with a 30 CV linear gradient of 2.5–15% buffer B. Fractions were analyzed by SDS-PAGE, and those containing *Ab*HisIE were pooled and dialyzed against 2 × 2 L of buffer C (50 mM HEPES pH 8.0, 250 mM NaCl), filtered through a 0.45 μM membrane and loaded onto a ZnCl_2_-charged 5 mL HisTrap FF column pre-equilibrated with buffer C. The column was washed with 10 CV of buffer C and the adsorbed proteins were eluted with a 20 CV linear gradient of 0–15% buffer D (50 mM HEPES pH 8.0, 250 mM NaCl, 50 mM imidazole). Fractions were analyzed by SDS-PAGE and those containing *Ab*HisIE were pooled, concentrated using a 10,000-MWCO ultrafiltration membrane, and loaded onto a HiPrep 26/60 Sephacryl S200 HR column equilibrated with buffer A. The column was washed with 1 CV of buffer A. Fractions were analyzed via SDS-PAGE and those containing *Ab*HisIE were pooled, concentrated using a 10,000-MWCO ultrafiltration membrane, aliquoted, and stored at −80 °C. The concentration of *Ab*HisIE was determined spectrophotometrically (NanoDrop) using a theoretical extinction coefficient (ε_280_) of 40,910 M^–1^ cm^–1^ (ProtParam tool – Expasy). The identity of the protein was confirmed via tryptic digest and LC–MS/MS analysis of the tryptic peptides performed by the University of St Andrews BSRC Proteomics and Mass Spectrometry facility.

For *Ab*HisE^domain^ purification, cells were resuspended in buffer A (50 mM HEPES pH 8.0, 500 mM NaCl, 10 mM imidazole) supplemented with 0.2 mg mL^–1^ lysozyme, 750 U BaseMuncher endonuclease, and half a tablet of EDTA-free Complete protease inhibitor cocktail. Cells were lysed in a cell disruptor (Constant systems) at 30 kpsi and centrifuged at 48,000 *g* for 30 min to remove cell debris. The supernatant was filtered through a 0.45 μm membrane and loaded onto a NiCl_2_-charged 5 mL HisTrap FF column pre-equilibrated with buffer A. The column was washed with 10 CV of buffer A, and adsorbed proteins were eluted with a 20 CV gradient of 0–100% buffer B (50 mM HEPES pH 8.0, 500 mM NaCl, 500 mM imidazole). Fractions were analyzed by SDS-PAGE and those containing *Ab*HisE^domain^ were pooled, mixed with TEVP (1 mg of TEVP to 15 mg of *Ab*HisE^domain^) and dialyzed against 2 × 2 L of buffer C (20 mM HEPES pH 7.5, 150 mM NaCl, 10% glycerol (v/v), 2 mM DTT) and against 1 × 2 L of buffer A. Samples were filtered through a 0.45 μm membrane and loaded onto a 5 mL HisTrap FF pre-equilibrated with buffer A. The column was washed with 10 CV of buffer A, and the eluate was collected, analyzed by SDS-PAGE, concentrated using a 10,000-MWCO ultrafiltration membrane, dialyzed against 2 × 2 L of buffer D (20 mM HEPES pH 8.0), aliquoted, and stored at −80 °C. The concentration of *Ab*HisE^domain^ was determined spectrophotometrically (NanoDrop) using an ε_280_ of 12,950 M^–1^ cm^–1^ (ProtParam tool – Expasy). The exact mass of the protein was determined via electrospray ionization mass spectrometry (ESI-MS) analysis by the University of St Andrews BSRC Proteomics and Mass Spectrometry facility.

### Syntheses of PRATP, PRAMP, and PRADP

For PRATP synthesis, a 10 mL reaction contained 8 μM *Ec*PRPPS, 15 μM *Pa*HisG_S_, 25 μM *Mt*PPase, 0.5 mM d-ribose-5-phosphate, and 0.75 mM ATP, 72 U mL^–1^*Sc*PK, 72 U mL^–1^*Sc*MK, 100 mM tricine pH 8.5, 100 mM KCl, 10 mM MgCl_2_, and 4 mM DTT. For PRAMP synthesis, a 10 mL reaction contained 15 μM *Ab*HisE^domain^ in addition to the aforementioned components. For PRADP synthesis, a 10 mL reaction contained 30 μM *Pa*HisG_S_, 25 μM *Mt*PPase, 12 mM ADP, 10 mM PRPP, 100 mM tricine pH 8.5, 100 mM KCl, 10 mM MgCl_2_, and 4 mM DTT. Reactions were incubated for 90 min at room temperature. Proteins were removed by passage through a 10,000-MWCO Vivaspin centrifugal concentrator and each filtrate was loaded onto a 20 mL HiTrap Q HP column (GE Healthcare) pre-equilibrated with water in a Bio-Rad NGC FPLC. The column was washed with 3 CV of water and 5 CV of either 6% or 18% solution B (1 M ammonium bicarbonate) for either PRAMP or PRATP purification, respectively, and with 1 CV of water and 5 CV of 10% solution B for PRADP. PRAMP was eluted with a 20-CV linear gradient of 6–24% solution B. PRATP was eluted with a 20-CV linear gradient of 18–30% solution B. PRADP was eluted with a 25-CV linear gradient of 10–30% solution B. Fractions exhibiting absorbance at 290 nm were pooled, lyophilized, and stored at −80 °C. The concentration of each compound was determined spectrophotometrically (NanoDrop) at 290 nm in 20 mM HEPES pH 7.5 (ε_290_ = 2800 M^–1^ cm^–1^).^23^

PRADP and PRATP were solubilized in water and loaded onto an Atlantis Premier BEH C_18_ AX column (2.1 × 100 mm, 1.7 μm) on a Waters ACQUITY UPLC system coupled to a Xevo G2-XS QToF mass spectrometer equipped with an ESI source. The UPLC mobile phase was (A) 10 mM ammonium acetate pH 6, (B) acetonitrile, and(C) 10 mM ammonium acetate pH 10. The following sequence was applied: 0–0.5 min at 90% (A) and 10% (B); 0.5–2.5 min step change from 90% (A) and 10% (B) to 50% (A), 10% (B) and 40% (C); 2.5–7 min re-equilibration to 90% (A) and 10% (B), the flow rate of 0.3 mL min^–1^. ESI data were acquired in negative mode with a capillary voltage of 2500 V. The source and desolvation gas temperatures of the mass spectrometer were 100 and 250 °C, respectively. The cone gas flow was 50 L h^–1^, whilst the gas flow was 600 L h^–1^. A scan was performed between 50 and 1200 *m/z*. A lockspray signal was measured and a mass correction was applied by collecting every 10 s, averaging 3 scans of 0.5 s each, using Leucine Enkephalin as a correction factor for mass accuracy.

PRAMP was solubilized in water and loaded onto a Premier BEH C_18_ column (2.1 × 50 mm, 1.7 μm) held at 40 °C on a Waters Arc HPLC system coupled to a QDa mass detector equipped with an ESI source. PRAMP was eluted with an isocratic mobile phase of 0.1% formic acid, 1% acetonitrile in water at a flow rate of 0.4 mL min^–1^. MS scans were performed followed by the selection of the desired ion for PRAMP. ESI data were acquired in negative mode with a capillary voltage of 800 V both as a mass range (50–1250) scan (cone voltage of 30 V) and single-ion (558) recording (cone voltage ramp of 30–100 V). The source and probe temperatures of the mass spectrometer were 120 and 600 °C, respectively.

### Detection of ProFAR by LC–MS

Reactions (500 μL) were prepared in 100 mM HEPES pH 7.5, 15 mM MgCl_2_, 16 μM *Ab*HisIE, and 135 μM PRATP. Control reactions lacked *Ab*HisIE. The reactions were incubated at room temperature for 1 h and passed through 10,000 MWCO Vivaspin centrifugal concentrators. ProFAR was detected exactly as described above for PRADP and PRATP detection by LC–MS.

### DSF-Based Thermal Denaturation of *Ab*HisIE and *Ab*HisE^domain^

DSF measurements (λ_ex_ = 490 nm, λ_em_ = 610 nm) were performed in 96-well plates on a Stratagene Mx3005p instrument. Reactions (50 μL) contained either 8.3 μM *Ab*HisIE (in the presence or absence of 50 μM PRATP) or 8.7 μM *Ab*HisE^domain^ in 100 mM HEPES pH 7.5 and 15 mM MgCl_2_. Invitrogen Sypro Orange (5×) was added to all wells. Controls lacked protein and were subtracted from the corresponding protein-containing samples. Thermal denaturation curves were recorded over a temperature range of 25–93 °C with increments of 1 °C min^–1^. Three independent measurements were carried out.

### *Ab*HisIE Direct and Continuous Assay Detecting ProFAR

Typically, initial rates were measured at 25 °C in 100 mM HEPES pH 7.5, 15 mM MgCl_2_, and 4 mM DTT, unless otherwise stated. Reactions (500 μL) were monitored for an increase in absorbance at 300 nm corresponding to the formation of ProFAR (Δε_300_ = 6700 M^–1^ cm^–1^ at pH 7.5)^9^ for 60 s in 1 cm path length quartz cuvettes (Hellma) in a Shimadzu UV-2600 spectrophotometer outfitted with a CPS unit for temperature control. Control reactions lacked *Ab*HisIE. The effect of added Zn^2+^ on activity was determined by measuring initial rates in the presence or absence of added 50 μM ZnCl_2_ and 13.5 μM PRATP, 12 mM MgCl_2_, and 20 nM *Ab*HisIE. The effect of added Mg^2+^ on activity was determined by measuring the initial rates in the presence of 0–24 mM MgCl_2_, 37 μM PRATP, and 20 nM *Ab*HisIE. The enzyme concentration dependence of the initial rate was determined in 100 mM HEPES pH 7.5, 12 mM MgCl_2_, and 4 mM DTT in the presence of 0–42 nM *Ab*HisIE and 37 μM PRATP. Progress curves of ProFAR formation from 40 μM PRATP were obtained in the presence of 21 nM *Ab*HisIE by monitoring the reaction for 1000 s.

### *Ab*HisIE Saturation Kinetics with PRATP and PRAMP

Initial rates of ProFAR formation were measured in the presence of 18 nM *Ab*HisIE, 100 mM HEPES pH 7.5, 15 mM MgCl_2_, 4 mM DTT, and varying concentrations of either PRATP (0–42 μM) or PRAMP (0–80 μM). Two independent measurements were carried out.

### *Ab*HisIE Saturation Kinetics in the Presence of Glycerol

Initial rates of ProFAR formation were measured in the presence of 18 nM *Ab*HisIE 100 mM HEPES pH 7.5, 15 mM MgCl_2_, 4 mM DTT, and varying concentrations of PRATP (0–42 μM) in the presence of 0–27% glycerol (v/v). Two independent measurements were carried out.

### Determination of PRATP ε_300nm_ at Different pH Values

The ε_300_ of PRATP at pH 7.0, 7.5, and 8.0 were determined by measuring the absorbance (NanoDrop) at 300 nm of known concentrations of PRATP in 100 mM HEPES (pH 7.0: 0.45, 0.91, and 1.8 mM; pH 7.5: 0.51, 1.0, and 2 mM; pH 8.0: 0.46, 0.92, and 1.8 mM). These known concentrations were determined independently via absorbance at 290 nm of a high-concentration stock solution of PRATP in 10 mM HEPES pH 7.5. This stock solution was in turn diluted into 100 mM HEPES at different pH values. The pH of the final PRATP solutions was measured to ensure that they stayed at pH 7.0, 7.5, and 8.0. Controls were measured by following the same dilution procedure in the absence of PRATP, and their absorbance at 300 nm was subtracted from each value with PRATP. The final values were then subtracted from the ε_300_ of ProFAR (8000 M^–1^ cm^–1^).^9^ to generate Δε_300_. Three independent measurements were carried out for each concentration at each pH.

### *Ab*HisIE Rates at pL 7.0, 7.5, and 8.0

The initial rates of ProFAR formation were measured in 100 mM HEPES pH 7.0, 7.5, and 8.0, 15 mM MgCl_2_, 4 mM DTT, and varying PRATP concentrations (2.3–37 μM for pH 7.0; 2.6–42 μM for pH 7.5; 1.4–40 μM for pH 8.0). *Ab*HisIE concentrations were 31, 18, and 7.5 nM for pH 7.0, 7.5, and 8.0, respectively. To ensure enzyme stability in the pH range, *Ab*HisIE was diluted in buffer at either pH 7.0 or 8.0 prior to activity assay at pH 7.5. The solvent deuterium kinetic isotope effects were determined by measuring the initial rates of ProFAR formation in 100 mM HEPES pD 7.0, 7.5, and 8.0 (pD = pH + 0.4),^24^ 15 mM MgCl_2_, 4 mM DTT, varying PRATP concentrations (2.3–59 μM for pD 7.0; 2.4–38 μM for pD 7.5; 2.6–48 μM for pD 8.0), and *Ab*HisIE concentrations of 186, 74, and 19 nM for pD 7.0, 7.5, and 8.0, respectively, in 99.5% D_2_O (v/v). The initial rates of ProFAR formation from varying concentrations of PRAMP (5–80 μM) were measured in 100 mM HEPES pL 7.5, and 8.0, 15 mM MgCl_2_, 4 mM DTT, and 18 nM *Ab*HisIE in either H_2_O or 99.5% D_2_O (v/v). Two independent measurements were carried out for each concentration at each pL.

### *Ab*HisIE and *Ab*HisE^domain^ Coupled Assay Detecting PP_i_

The pyrophosphohydrolase activities of the *Ab*HisIE and *Ab*HisE^domain^ were independently assessed via the EnzCheck Pyrophosphate Assay kit.^25^ The initial rates were measured in the presence of 100 mM HEPES pH 7.5, 15 mM MgCl_2_, 4 mM DTT, 200 μM 2-amino-6-mercapto-7-methylpurine ribonucleoside (MESG), 1 U mL^–1^ of purine nucleoside phosphorylase (PNP), and 0.03 U mL^–1^ of PPase. The reactions (500 μL) were monitored for the increase in absorbance at 360 nm (Δε_360nm_ = 11,000 M^–1^ cm^–1^) upon phosphorolysis of MESG to 2-amino-6-mercapto-7-methylpurine at 25 °C for 60 s in 1 cm path length cuvettes (Hellma) using a Shimadzu UV-2600 spectrophotometer outfitted with a CPS unit for temperature control. The initial rates of PP_i_ formation were measured in the presence of 8.4 μM PRATP and either 0–6.4 nM *Ab*HisIE or 0–20 nM *Ab*HisE^domain^. *Ab*HisIE substrate saturation curves were collected with varying concentrations of PRATP (0–16 μM) and 3.2 nM *Ab*HisIE. Controls lacked, in turn, *Ab*HisIE or *Ab*HisE^domain^, PRATP, and PPase. Two independent measurements were carried out.

### Pre-Steady-State Kinetics

The approach to steady-state for ProFAR formation by *Ab*HisIE under multiple-turnover conditions at 25 °C was carried out by monitoring the increase in absorbance at 300 nm for 0.2 s in an Applied Photophysics SX-20 stopped-flow spectrophotometer outfitted with a 5 μL mixing cell (0.5 cm path length and 0.9 ms dead time) and a circulating water bath. Each syringe contained 100 mM HEPES pH 7.5, 15 mM MgCl_2_, and 4 mM DTT. In addition, in the first experiment, one syringe contained 10 μM *Ab*HisIE and the other, 50 μM PRATP. In the second experiment, one syringe contained 20 μM *Ab*HisIE and the other, 100 μM PRATP. The reaction was triggered by rapidly mixing 55 μL from each syringe. In each experiment, 4 traces were collected with 3000 data points per trace. Controls lacked enzyme.

### *Ab*HisIE Inhibition by PRADP

The initial rates of ProFAR formation from PRATP were measured at 25 °C in 100 mM HEPES pH 7.5, 15 mM MgCl_2_, and 4 mM DTT in the presence of 18 nM *Ab*HisIE and varying concentrations of PRADP (0–160 μM). Alternatively, the initial rates of ProFAR formation from 20 μM PRAMP were measured in the presence of 18 nM *Ab*HisIE and either 0 or 268 μM PRADP. Incubation of 42 μM PRADP with either 0 or 18 nM *Ab*HisIE under ProFAR formation assay conditions for 30 min revealed no increase in absorbance at 300 nm. The incubation of 40 μM PRADP with either 0 or 18 nM *Ab*HisIE under PPi formation coupled assay conditions for 5 min detected no increase in absorbance at 360 nm. For comparison, under corresponding assay conditions, ProFAR is detected in less than 10 s from 37 μM PRATP and 10 nM *Ab*HisIE, and PPi, in less than 10 s from 8.4 μM PRATP and 1.6 nM *Ab*HisIE.

### Analysis of Kinetics Data

Kinetics data were analyzed by the non-linear regression function of SigmaPlot 14.0 (SPSS Inc.). Data points and error bars represent mean ± SEM, and kinetic and equilibrium constants are given as mean ± fitting error. Substrate saturation curves were fitted to eq 1, solvent viscosity effects were fitted to eq 2, solvent deuterium kinetic isotope effects were fitted to eq 3, and inhibition data were fitted to eq 4. In eqs 1–4, *v* is the initial rate, *k*_cat_ is the steady-state turnover number, *K*_M_ is the Michaelis constant, *E*_T_ is total enzyme concentration, *S* is the concentration of substrate, *k*_0_ and *k*_η_ are the rate constants in the absence and presence of glycerol, respectively, η_rel_ is the relative viscosity of the solution, *m* is the slope, *F*_i_ is the fraction of deuterium label, *E*_*k*_cat_/*K*_M__ and *E*_*k*_cat__ are the solvent isotope effect minus 1 on *k*_cat_/*K*_M_ (^D2O^(*k*_cat_/*K*_M_)), and *k*_cat_ (^D2O^*k*_cat_) respectively, *v*_i_ and *v*_0_ are the initial rates in the presence and absence of inhibitor, respectively, IC_50_ is the half-maximal inhibitory concentration, and *h* is the Hill coefficient. ^D2O^(*k*_cat_/*K*_M_) and ^D2O^*k*_cat_ at pL 7.0 and 7.5 were also calculated as the ratios of the relevant rate constants in H_2_O and D_2_O.
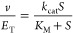
1
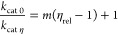
2
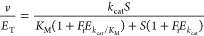
3
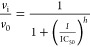
4

## Results and Discussion

### Purification, Biophysical, and Biochemical Characterization of *Ab*HisIE and *Ab*HisE^domain^

These results, including Figures S1–S14, are described and discussed in Supporting Information.

### Steady-State Kinetic Parameters for *Ab*HisIE with PRATP

**Figure 1:**
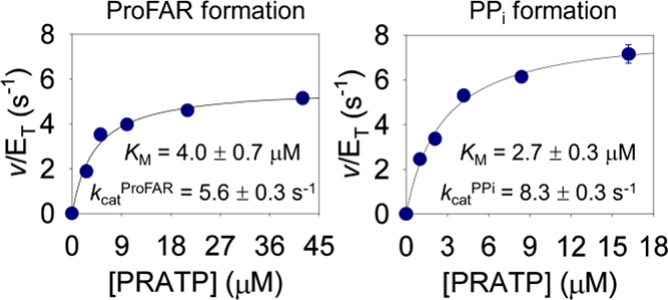
Substrate saturation curves and associated apparent steady-state kinetic parameters for *Ab*HisIE-catalyzed ProFAR formation and PPi formation. Lines are best fit to eq 1. A Student’s t-test indicated *k*_cat_^ProFAR^ and *k*_cat_^PPi^ are statistically different (*p* < 0.025).

To uncover which of the two reactions, i.e., either pyrophosphohydrolase-catalyzed or cyclohydrolase-catalyzed, is rate-limiting, the steady-state kinetic parameters were determined based on the detection of ProFAR and PP_i_. The *Ab*HisIE overall reaction displayed Michaelis–Menten kinetics when PRATP concentration was varied and either ProFAR or PPi formation was measured (Figure 1). When ProFAR formation was measured, fitting the data to eq 1 yielded an apparent steady-state catalytic constant (*k*_cat_^ProFAR^) and apparent Michaelis constant (*K*_M_) shown in Figure 1, resulting in *k*_cat_^ProFAR^/*K*_M_ of (1.4 ± 0.3) × 10^6^ M^–1^ s^–1^. The values obtained here are comparable to those reported for ProFAR formation by *Methanococcus vannielii* HisI (*k*_cat_ = 4.1 s^–1^; *k*_cat_/*K*_M_ = 4.1 × 10^5^ M^–1^ s^–1^) and *M. thermoautotrophicum* HisI (*k*_cat_ = 8 s^–1^; *k*_cat_/*K*_M_ = 6 × 10^5^ M^–1^ s^–1^), but in the case of these monofunctional HisI, the substrate was PRAMP.^6,9^ When PP_i_ formation was monitored, data fitting to eq 1 yielded apparent *k*_cat_^PPi^ and apparent *K*_M_ as depicted in Figure 1, leading to *k*_cat_^PPi^/*K*_M_ of (3.1 ± 0.4) × 10^6^ M^–1^ s^–1^.

**Scheme 2:**
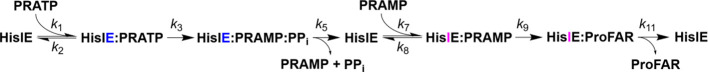
The Minimum Kinetic Sequence for the *Ab*HisIE-catalyzed Reaction

Assuming catalytically independent active sites in the HisE- and HisI-like domains of *Ab*HisIE, and PRAMP binding to the HisI-like domain in a bimolecular step, a minimum kinetic sequence depicting flux through the bifunctional enzyme reaction from PRATP can be summarized in Scheme 2, with the release of PRAMP and PP_i_ from the HisE-like domain combined in one step (*k*_5_) since the order (if any) of product release is not known. Besides the irreversibility of the two hydrolytic steps (*k*_3_ and *k*_9_), product release steps (*k*_5_ and *k*_11_) are presumed irreversible under initial-rate conditions in the absence of added products.^26^

The makeup of the steady-state kinetic parameters shown in Figure 1 will differ depending on which product is being measured, even though only PRATP concentration is varied. When PP_i_ is detected, *k*_cat_^PPi^, at saturating levels of PRATP, and *k*_cat_^PPi^/*K*_M_, at PRATP levels approaching zero, are defined by simple expressions as in eqs 5 and 6, respectively. On the other hand, when ProFAR is detected, the corresponding steady-state parameters are more complex. Increasing concentrations of PRATP must eventually lead to saturation of the HisI-like domain active site with PRAMP, as seen from the saturation kinetics obtained when the final product is monitored (Figure 1), and *k*_cat_^ProFAR^ is given by eq 7. As PRATP levels approach zero, so do PRAMP levels, and *k*_cat_^ProFAR^/*K*_M_ is defined by eq 8 (eqs 5–8 derived according to Cleland’s Net Rate Constant method,^26^ see Supporting Information for details).
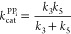
5
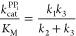
6
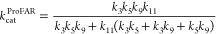
7
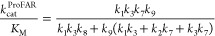
8

Given the complexity of the *Ab*HisIE catalytic sequence and the statistically different yet comparable magnitudes of *k*_cat_^PPi^ and *k*_cat_^ProFAR^, it is possible that both reactions are kinetically relevant to *k*_cat_^ProFAR^, with the cyclohydrolase reaction making a larger contribution.

### Steady-State Kinetic Parameters for *Ab*HisIE with PRAMP

**Figure 2:**
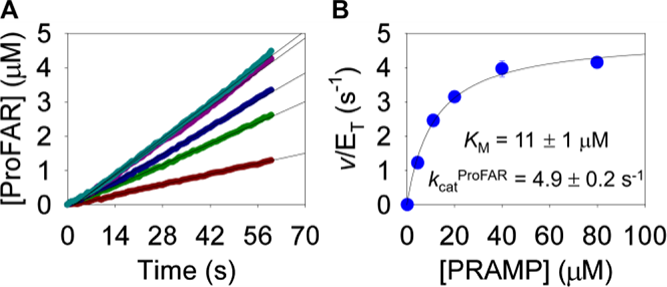
Kinetic competence of PRAMP. (A) Time courses of ProFAR formation from different PRAMP concentrations. Thick lines are mean traces from two independent measurements; thin black lines are linear regressions of the data. (B) PRAMP saturation curve and associated apparent steady-state kinetic parameters for *Ab*HisIE-catalyzed ProFAR formation. The line is best fit to eq 1.

To determine if PRAMP can bind the HisI-like domain from bulk solvent and to test its kinetic competence as a substrate for the cyclohydrolase-catalyzed reaction, steady-state kinetic parameters for ProFAR formation were determined with PRAMP as the varying substrate, bypassing the necessity for the pyrophosphohydrolase-catalyzed reaction altogether. ProFAR formation was readily detected (Figure 2A), and the reaction followed Michaels–Menten kinetics, with data fitting to eq 1 yielding steady-state kinetic parameters displayed in Figure 2B. The similar *k*_cat_^ProFAR^ values when either PRATP or PRAMP was the substrate suggest PRAMP can bind the HisI-like domain of *Ab*HisIE from bulk solvent and the cyclohydrolase-catalyzed reaction limits the overall rate of the bifunctional catalytic cycle. The *k*_cat_^ProFAR^/*K*_M_ for PRAMP of (4.4 ± 0.4) × 10^5^ M^–1^ s^–1^ is 3-fold lower than the *k*_cat_^ProFAR^/*K*_M_ for PRATP, which speaks against diffusion in and out of bulk solvent as the main path for PRAMP transfer from the first active site to the second, favoring the hypothesis that *Ab*HisIE catalysis involves proximity channeling.^27,28^

### Solvent Viscosity Effects on *Ab*HisIE-Catalyzed ProFAR Formation

**Figure 3:**
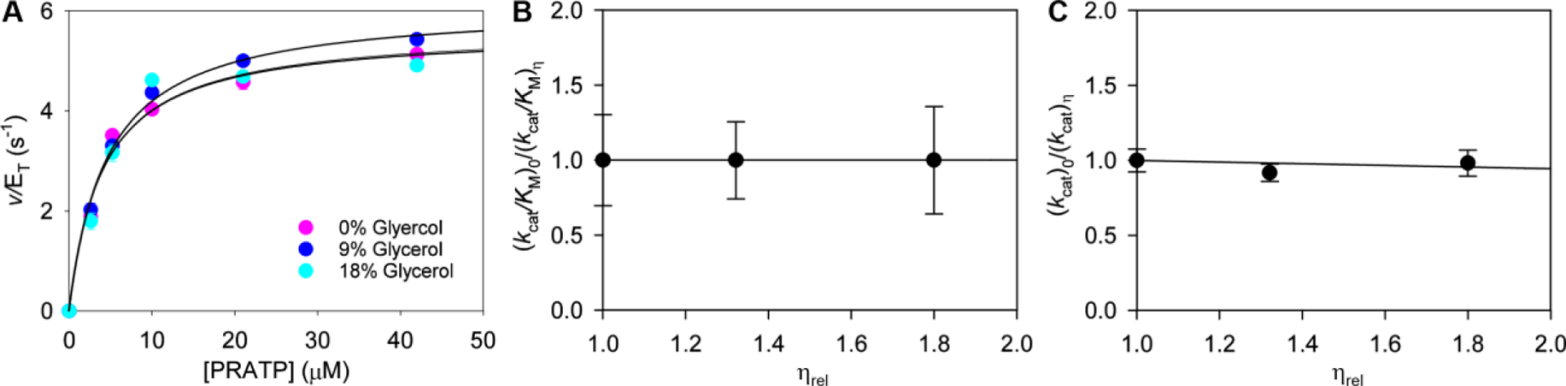
Solvent viscosity effects on *Ab*HisIE-catalyzed reaction. (A) Substrate saturation curves for *Ab*HisIE-catalyzed ProFAR formation in the presence and absence of glycerol. Lines are best fit to eq 1. (B) Solvent viscosity effects on *k*_cat_^ProFAR^/*K*_M_. (C) Solvent viscosity effects on *k*_cat_^ProFAR^. Lines are best fit to eq 2.

In order to evaluate whether or not diffusional steps involving either substate binding or product release limit the rate of *Ab*HisIE-catalyzed ProFAR formation from PRATP, solvent viscosity effects were determined by measuring reaction rates at different concentrations of the microviscogen glycerol (Figure 3A and Table S1). Plots of *k*_cat_^ProFAR^/*K*_M_ ratios versus relative viscosity (Figure 3B) and *k*_cat_^ProFAR^ ratios versus relative viscosity (Figure 3C) produced slopes of 0 and −0.01, respectively. These data indicate neither PRATP and PRAMP binding to nor PRAMP, PP_i_, and ProFAR release from *Ab*HisIE is rate-limiting in the reaction.^29^ This contrasts with the first enzyme of histidine biosynthesis, ATPPRT, where significant solvent viscosity effects on *k*_cat_ revealed diffusion of the product from the enzyme to be rate-limiting.^30^

### Solvent Deuterium Isotope Effects on *Ab*HisIE-Catalyzed ProFAR Formation

**Figure 4:**
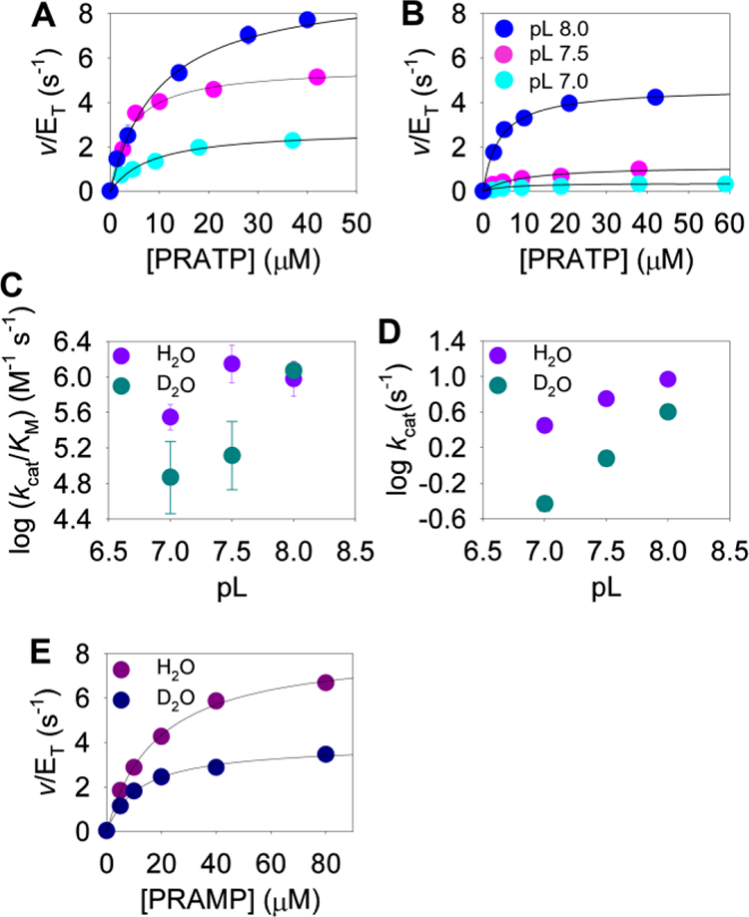
*Ab*HisIE kinetics in H_2_O and D_2_O. (A) PRATP saturation curves at pH 7.0, 7.5, and 8.0. Lines are best fit to eq 1. (B) PRATP saturation curves at pD 7.0, 7.5, and 8.0. Lines are best fit to eq 3. (C) Dependence of *k*_cat_^ProFAR^/*K*_M_ on pL. (D) Dependence of *k*_cat_^ProFAR^ on pL. (E) PRAMP saturation curves at pL 7. Lines are best fit to eq 1 for data in H_2_O and to eq 3 for data in D_2_O. L denotes either H or D.

Solvent deuterium isotope effects, defined as the ratio of rate constants (kinetic isotope effects) or equilibrium constants (equilibrium isotope effects) for reactions taking place in H_2_O and D_2_O, can inform on rate-limiting proton-transfer steps in enzymatic reactions.^31^ To uncover potential rate-liming proton-transfer steps in the *Ab*HisIE reaction, a solvent deuterium isotope effect study was undertaken. Because the presence of D_2_O can increase the p*K*_a_ of kinetically relevant ionizable groups by ∼0.5, solvent isotope effects would ideally be measured in a pH-independent region of a pH-rate profile.^32^ In the case of *Ab*HisIE, this is further complicated by the fact that, while ProFAR absorbance is pH independent above pH 5,^33^ PRATP and PRAMP absorbance at 300 nm is pH-dependent (PRATP and PRAMP have identical absorbance spectra in this region),^23^ which would shift the Δε_300_ from its value at pH 7.5.^9^ Hence, Δε_300_ was determined for the conversion of PRATP to ProFAR under initial-rate conditions by measuring ε_300_ for PRATP at pH 7.0, 7.5, and 8.0 (Figure S14) and subtracting each value from the ε_300_ for ProFAR^9^ (Table S2). Importantly, the Δε_300_ for ProFAR at pH 7.5 obtained by this method (6690 M^–1^ cm^–1^) is within 0.15% of the published value, lending confidence to the approach. With PRATP as the substrate, both *k*_cat_^ProFAR^/*K*_M_ and *k*_cat_^ProFAR^ increased as the pL increased from 7.0 to 8.0, although *k*_cat_^ProFAR^/*K*_M_ seemed to peak at pH 7.5 when the reaction took place in H_2_O (Figure 4), indicating that deprotonation of one or more groups increases the reaction rate.

Solvent deuterium isotope effect measurement at a plateau region of the pH-rate profile could not be accomplished here. Therefore, caution must be wielded to interpret the magnitudes of the isotope effects reported in Table 1, especially for ^D2O^(*k*_cat_^ProFAR^/*K*_M_) where the propagated experimental uncertainties were sizable owing to the uncertainties in the second-order rate constants obtained in D_2_O (Figure 4C). As a trend, ^D2O^(*k*_cat_^ProFAR^/*K*_M_) and ^D2O^*k*_cat_^ProFAR^ decreased as the pL increased, suggesting proton-transfer steps accompany steps contributing less to the observed reaction rate as the pH increases. It should be noted that *k*_cat_^ProFAR^/*K*_M_ and *k*_cat_^ProFAR^ varied by a maximum of 4- and 3.4-fold, respectively, across the pH range. This is particularly relevant for ^D2O^(*k*_cat_^ProFAR^/*K*_M_) and ^D2O^*k*_cat_^ProFAR^ at pL 7.5, which are higher than what could be accounted for by pL changes alone.

**Table 1:** Solvent Deuterium Isotope Effects and pH Effects on Steady-State Parameters for *Ab*HisIE-Catalyzed ProFAR Formation

parameter	pL 7.0	pL 7.5	pL 8.0
*k*_cat_^ProFAR^ (s^–1^)	2.8 ± 0.2	5.6 ± 0.3	9.6 ± 0.4
*K*_M_ (μM)	8 ± 1	4.0 ± 0.7	10 ± 2
*k*_cat_^ProFAR^/*K*_M_ (M^–1^ s^–1^)	(3.5 ± 0.5) × 10^5^	(1.4 ± 0.3) × 10^6^	(9.6 ± 0.9) × 10^5^
^D2O^(*k*_cat_^ProFAR^/*K*_M_)	12 ± 4^a^	12 ± 5^a^	0.8 ± 0.1
^D2O^*k*_cat_^ProFAR^	6.8 ± 0.6	4.9 ± 0.6	2.0 ± 0.1

a Values calculated as the ratios of the relevant kinetic parameters in H_2_O and D_2_O.

The reactions taking place in the pyrophosphohydrolase and cyclohydrolase active sites are proposed to involve nucleophilic attack by Mg^2+^- and Zn^2+^-activated water molecules, respectively,^5,7,10^ and it is reasonable to assume water coordination by the metal will be in equilibrium in the free *Ab*HisIE. As Mg^2+^-activated^34,35^ and Zn^2+^-activated^32^ H_2_O/D_2_O can have inverse fractionation factors (ϕ_M-OL_ ∼ 0.7–0.9), the observed ^D2O^(*k*_cat_^ProFAR^/*K*_M_) will be equal to the product of an inverse equilibrium solvent isotope effect (^D2O^*K*_eq_ < 1) and any subsequent solvent kinetic isotope effects. This means the normal kinetic isotope effect portion of ^D2O^(*k*_cat_^ProFAR^/*K*_M_) has a larger value than what was observed, for instance, at pH 7.0 and 7.5, likely the result of more than one proton in flight during a rate-limiting step for *k*_cat_^ProFAR^/*K*_M_. At pH 8.0, ^D2O^(*k*_cat_^ProFAR^/*K*_M_) becomes modestly inverse, probably reflecting an inverse ^D2O^*K*_eq_ on metal-water coordination once a slow proton-transfer step at lower pH becomes fast at this higher pH. The proposed catalytic mechanism for cyclodrolysis of PRAMP^10^ is reminiscent of that proposed for the Zn^2+^-dependent metalloenzyme AMP deaminase, where an inverse ^D2O^(*k*_cat_^ProFAR^/*K*_M_) of ∼0.7 was also reported, followed by a proton inventory implicating at least two proton transfers in a rapid equilibrium step involving Zn^2+^-water coordination.^36^ While ^D2O^*k*_cat_^ProFAR^ decreases at pH 8.0, it remains normal and significant, indicating protonation steps reporting on *k*_cat_^ProFAR^/*K*_M_ and *k*_cat_^ProFAR^ are separated by an irreversible step. Importantly, at all pHs tested, at least one proton is in flight during the rate-limiting step for *k*_cat_^ProFAR^, which our results indicate has larger contribution from the cyclohydrolysis reaction.

At pH 7.5, when PRAMP was employed as a substrate to bypass the pyrophospholysis reaction, the ^D2O^(*k*_cat_^ProFAR^/*K*_M_) was only 1.4 ± 0.1 (Figure 4E). This suggests a large portion of the ^D2O^(*k*_cat_^ProFAR^/*K*_M_) observed with PRATP as a substrate reports on PRATP pyrophosphohydrolysis. Assuming, hypothetically, the Zn^2+^-bound water molecule responsible for the cyclohydrolysis of PRAMP would induce a ^D2O^*K*_eq_ of ∼0.7 (based on common fractionation factors attributed to Zn^2+^-bound water),^32^ the kinetic isotope effect portion of the ^D2O^(*k*_cat_^ProFAR^/*K*_M_) with PRAMP as a substrate would have a magnitude of ∼2.^32^ This also suggests a ^D2O^(*k*_cat_^ProFAR^/*K*_M_) of ∼8.5 originating in the PRATP pyrophosphohydrolysis reaction, probably involving more than one proton in flight. ^D2O^*k*_cat_^ProFAR^ was 2.1 ± 0.1 (Figure 4E) with PRAMP as a substrate. This suggests at least one proton is in flight during the rate-limiting step for *k*_cat_^ProFAR^ from the *Ab*HisIE:PRAMP complex, and this contributes about half the overall ^D2O^*k*_cat_^ProFAR^ from the *Ab*HisIE:PRATP complex.

### Lag and Burst Phases of ProFAR Formation from PRATP

**Figure 5:**
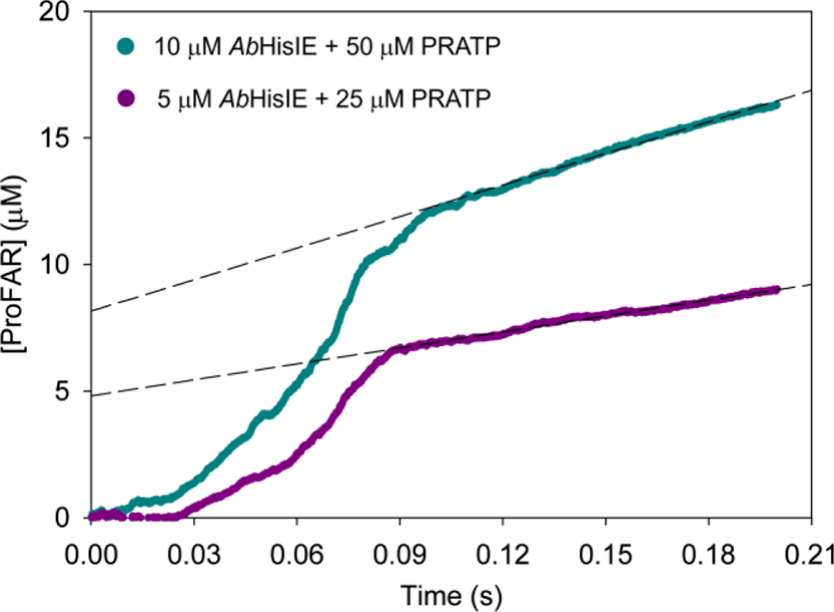
Rapid kinetics of approach to a steady state of *Ab*HisIE-catalyzed ProFAR production from PRATP. The dashed lines are linear regressions of the linear phases.

To uncover additional information on rate-limiting steps of *Ab*HisIE-catalyzed ProFAR synthesis from PRATP, the approach to steady state was monitored upon rapid mixing of *Ab*HisIE and PRATP at pH 7.5 (Figure 5). The curves could not be fitted to an equation because, even though PRATP concentration was in 5-fold excess to enzyme concentration, PRAMP concentration was not, as it was being formed in situ. Qualitatively, the data are interpreted as follows. As PRATP concentrations used were at least 10-fold the *K*_M_ obtained when PP_i_ formation was assayed, the lag phase, which is shorter at the higher enzyme concentration, reflects mostly the PRAMP formation rate from the nearly saturated HisE active site of *Ab*HisIE, with no appreciable formation of ProFAR. As ProFAR production progresses, eventually the HisI-like active site is nearly saturated by PRAMP, leading to ProFAR formation resembling a burst that precedes the steady-state reaction. Supporting this interpretation, linear regressions of the linear phases yielded apparent steady-state rate constants of 4.2 ± 0.1 and 4.16 ± 0.08 s^–1^, in reasonable agreement with *k*_cat_^ProFAR^. Furthermore, the *y*-axis intercepts of the linear regressions indicating the concentrations of on-enzyme ProFAR formed in the burst phase, 4.8 ± 0.2 and 8.2 ± 0.3 μM, approach the corresponding *Ab*HisIE concentrations. However, the *k*_cat_^PPi^ of 8.3 s^–1^ would allow only ∼0.75 turnovers in ∼0.09 s, the apparent time required to saturate the HisI active site with PRAMP (Figure 5). Even if all *Ab*HisIE is bound to PRATP, only ∼3.75 and ∼7.5 μM of free PRAMP would be produced from 5 and 10 μM *Ab*HisIE, respectively, in 0.09 s, concentrations which are below the PRAMP *K*_M_ of 11 μM. This suggests the preferred pathway for the transfer of PRAMP from the pyrophosphohydrolase domain to the cyclohydolase domain avoids significant diffusion into bulk solvent. Too short a lag time in consecutive reactions to allow the intermediate to accumulate enough into bulk solvent before rebinding to the next active site has been invoked as characteristic of substrate channeling.^27^ A pre-steady-state burst was also observed in ATPPRT catalysis,^30,37^ and product release was shown to be rate-limiting based on solvent viscosity effects on *k*_cat_.^30^

### PRADP Inhibits *Ab*HisIE-Catalyzed Pyrophosphorolysis

**Figure 6:**
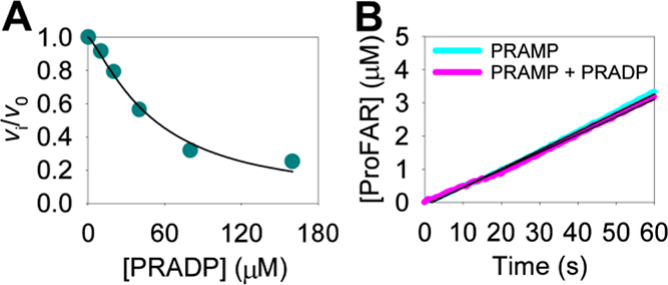
*Ab*HisIE inhibition by PRADP. (A) Dose-dependence curve of *Ab*HisIE-catalyzed ProFAR formation from PRATP in the presence of PRADP. The line is the best fit to eq 4. (B) Initial rates of *Ab*HisIE-catalyzed ProFAR formation from PRAMP in the presence and absence of PRADP. Thin black lines are linear regressions of the traces which produced initial rates of 0.0565 ± 0.0004 and 0.0534 ± 0.0004 μM s^–1^ in the absence and presence of PRADP.

ADP can replace ATP as a substrate of ATPPRT, which generates PRADP.^30^*Ab*HisIE, however, failed to produce ProFAR, PP_i_, or P_i_ when PRADP replaced PRATP as a substrate. As PRADP is a close structural analogue of both PRAMP and PRATP, we tested whether it might act as an *Ab*HisIE inhibitor. PRADP inhibited *Ab*HisIE-catalyzed ProFAR formation from PRATP in a dose-dependent manner (Figure 6A), and data fitting to eq 4 yieded an IC_50_ of 52 ± 4 μM. However, even 268 μM PRADP could not inhibit *Ab*HisIE-catalyzed ProFAR formation from 20 μM PRAMP (Figure 6B). This suggests that PRADP binds to the HisE-like domain of *Ab*HisIE and inhibits pyrophosphohydrolysis of PRATP to PRAMP. The β-phosphate group of PRADP might prevent its binding to the HisI-like domain of *Ab*HisIE, allowing ProFAR to form unincumbered from PRAMP directly. This provides further evidence of how independently the two active sites are able to operate and demonstrates the probable channeling of PRAMP does not involve a tunnel through the protein connecting the two active sites.^28^ A protein tunnel shielded from bulk solvent was also disfavoured as a connection between the two domains based on crystal structures of HisIE orthologues,^7,8^ and cannot be readily envisioned from our AlphaFold-based structural model (Figure S4A).

### Implications for *Ab*HisIE Catalysis

**Scheme 3:**
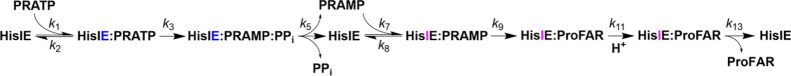
Expanded Kinetic Sequence for the *Ab*HisIE-Catalyzed Reaction

The presence of a burst preceding the steady state indicates a step following adenine ring-opening limits *k*_cat_^ProFAR^. While this is commonly interpreted as product release being rate-limiting,^30,38^ the lack of solvent viscosity effects on *k*_cat_^ProFAR^ rules out slow diffusion of ProFAR from *Ab*HisIE.^29^ Moreover, the sizable ^D2O^*k*_cat_^ProFAR^ means at least one proton transfer is associated with this step.^31,32^ In addition, it is clear from steady-state and pre-steady-state kinetic analyses that *k*_cat_^PPi^ is not large enough to allow PRAMP to accumulate into the bulk solvent at the levels required to saturate the cyclohydrolase active site, and some form of proximity channeling^28^ must be the preferred means of PRAMP transfer. Based on the inhibition of the pyrophosphohydrolase activity but not the cyclohydrolase activity by PRADP, channeling does not involve tunneling through the protein, and probably still involves bimolecular binding of PRAMP to the cyclohydrolase active site being faster than diffusion into bulk solvent. Hence, the catalytic cycle depicted in Scheme 2 must be expanded to include preferential partition of newly synthesized PRAMP toward binding the HisI-like active site as opposed to diffusion into bulk water, and at least a slow unimolecular step (*k*_11_) involving a proton transfer following adenine ring opening but preceding ProFAR release (Scheme 3). This might be, for instance, a proton-transfer-linked conformational change that triggers ProFAR dissociation.

In this revised catalytic sequence, *k*_cat_^ProFAR^ for ProFAR synthesis from PRATP is given by eq 9 (see Supporting Information for details). It should be pointed out that the rate constants in Schemes 2 and 3 do necessarily represent microscopic rate constants governing elementary steps, but potentially macroscopic rate constants^38^ defining the minimum catalytic path from PRATP to ProFAR.
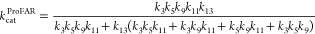
9

Metabolic advantages associated with channeling, such as increased flux through a biosynthetic pathway and protection of intermediates from the action of enzymes external to the pathway,^28^ may have favored the evolution of bifunctionality in *Ab*HisIE. It should be pointed out, however, that simple fusion of enzymes is neither sufficient nor required to ensure substrate channeling, as exemplified by the lack of channeling in the multifunctional AROM complex^39^ and the presence of channeling in the monofunctional proteins constituting the purinosome.^40^ Another potential advantage of gene fusion includes a fixed ratio of gene products for a set of consecutive reactions.^41^ Any fitness advantage associated with a bifunctional HisIE must be organism-specific, since other bacteria, such as *M. tuberculosis* have separate genes encoding monofunctional HisE and HisI.^3,5^ Future kinetic characterization of the *Ab*HisE^domain^ will elucidate how much, if any, of the catalytic ability of this domain is compromised by the loss of the HisI-like domain.

The inability of *Ab*HisIE to utilize PRADP as a substrate for either of its reactions is somewhat surprising with regards to its pyrophosphohydrolase activity. Other members of the α-helical NTP pyrophosphohydrolase superfamily, to which the HisE-like domain of *Ab*HisIE belongs, such as protozoan dUTPases, can efficiently hydrolyze both dUTP, releasing PP_i_, and dUDP, releasing P_i_, to dUMP.^42^ Unlike what its three-dimensional fold would predict, the pyrophosphohydrolase specificity of *Ab*HisIE seems reminiscent of trimeric all-β dUTPases, which cannot hydrolyze dUDP.^43^ In trimeric dUTPases, dUDP acts as a competitive inhibitor, sitting in the active site in the same orientation non-hydrolysable dUTP analogues do, and crystal structures of these enzymes in complex with dUDP shed light on how catalysis proceeds.^44,45^ Given their structural similarities, PRADP presumably acts as a competitive inhibitor against PRATP, and it could prove useful for obtaining a crystal structure of *Ab*HisIE, or other HisE enzymes, with a substrate analogue bound in the active site to furnish insight into the catalytic mechanism.

## Supporting Information Available

Additional results and discussion on the characterization of *Ab*HisIE (PDF)

## Abbreviations

PRPP: 5-phospho-α-D-ribosyl-1-pyrophosphate
PRATP: *N*^1^-(5-phospho-β-D-ribosyl)-ATP
ATPPRT: ATP phosphoribosyltransferase
HisE: phosphoribosyl-ATP pyrophosphohydrolase
PP_i_: pyrophosphate
HisI: phosphoribosyl-AMP cyclohydrolase
phosphoribosyl-ATP: pyrophosphohydrolase/phosphoribosyl-AMP cyclohydrolase
PRAMP: *N*^1^-(5-phospho-β-D-ribosyl)-AMP
ProFAR: *N*-(5′-phospho-D-ribosylformimino)-5-amino-1-(5″-phospho-D-ribosyl)-4-imidazolecarboxamide
*Ab*ATPPRT: *A. baumannii* ATPPRT
*Ab*HisIE: *A. baumannii* HisIE
LC–MS: liquid chromatography-mass spectrometry
DSF: differential scanning fluorimetry
*Ab*HisE^domain^: truncated *Ab*HisIE containing only the HisE-like domain
*k*_cat_: steady-state catalytic constant
*K*_M_: Michaelis constant

## References

[ref1] Ames B. N.; Martin R. G.; Garry B. J. The First Step Of Histidine Biosynthesis. J. Biol. Chem. 1961, 236, 2019–2026. 10.1016/S0021-9258(18)64123-7. 13682989

[ref2] Wang N.; Ozer E. A.; Mandel M. J.; Hauser A. R. Genome-Wide Identification Of Acinetobacter Baumannii Genes Necessary For Persistence In The Lung. mBio 2014, 5, e01163–e01114. 10.1128/mBio.01163-14. 24895306 PMC4049102

[ref3] Alifano P.; Fani R.; Lió P.; Lazcano A.; Bazzicalupo M.; Carlomagno M. S.; Bruni C. B. Histidine Biosynthetic Pathway And Genes: Structure, Regulation, And Evolution. Microbiol. Rev. 1996, 60, 44–69. 10.1128/mr.60.1.44-69.1996. 8852895 PMC239417

[ref4] Martin R. G.; Berberich M. A.; Ames B. N.; Davis W. W.; Goldberger R. F.; Yourno J. D. Enzymes And Intermediates Of Histidine Biosynthesis In *Salmonella Typhimurium*. Methods Enzymol. 1971, 17, 3–44. 10.1016/0076-6879(71)17003-6.

[ref5] Javid-Majd F.; Yang D.; Ioerger T. R.; Sacchettini J. C. The 1.25 Å Resolution Structure Of Phosphoribosyl-ATP Pyrophosphohydrolase From *Mycobacterium Tuberculosis*. Acta Crystallogr. D Biol. Crystallogr. 2008, 64, 627–635. 10.1107/S0907444908007105. 18560150 PMC2631106

[ref6] Sivaraman J.; Myers R. S.; Boju L.; Sulea T.; Cygler M.; Jo Davisson V.; Schrag J. D. Crystal Structure Of *Methanobacterium Thermoautotrophicum* Phosphoribosyl-AMP Cyclohydrolase HisI. Biochemistry 2005, 44, 10071–10080. 10.1021/bi050472w. 16042384

[ref7] Wang Y.; Zhang F.; Nie Y.; Shang G.; Zhang H. Structural Analysis Of *Shigella Flexneri* Bi-Functional Enzyme HisIE In Histidine Biosynthesis. Biochem. Biophys. Res. Commun. 2019, 516, 540–545. 10.1016/j.bbrc.2019.06.099. 31235255

[ref8] Witek W.; Sliwiak J.; Ruszkowski M. Structural And Mechanistic Insights Into The Bifunctional HISN2 Enzyme Catalyzing The Second And Third Steps Of Histidine Biosynthesis In Plants. Sci. Rep. 2021, 11, 9647 10.1038/s41598-021-88920-2. 33958623 PMC8102479

[ref9] D’Ordine R. L.; Klem T. J.; Davisson V. J. N1-(5’-Phosphoribosyl)Adenosine-5’-Monophosphate Cyclohydrolase: Purification And Characterization Of A Unique Metalloenzyme. Biochemistry 1999, 38, 1537–1546. 10.1021/bi982475x. 9931020

[ref10] D’Ordine R. L.; Linger R. S.; Thai C. J.; Davisson V. J. Catalytic Zinc Site And Mechanism Of The Metalloenzyme PR-AMP Cyclohydrolase. Biochemistry 2012, 51, 5791–5803. 10.1021/bi300391m. 22741521 PMC3427388

[ref11] Dwivedy A.; Ashraf A.; Jha B.; Kumar D.; Agarwal N.; Biswal B. K. De Novo Histidine Biosynthesis Protects *Mycobacterium Tuberculosis* From Host IFN-Γ Mediated Histidine Starvation. Commun. Biol. 2021, 4, 410 10.1038/s42003-021-01926-4. 33767335 PMC7994828

[ref12] Martínez-Guitián M.; Vázquez-Ucha J. C.; Álvarez-Fraga L.; Conde-Pérez K.; Lasarte-Monterrubio C.; Vallejo J. A.; Bou G.; Poza M.; Beceiro A. Involvement Of HisF In The Persistence Of *Acinetobacter Baumannii* During A Pneumonia Infection. Front. Cell Infect. Microbiol. 2019, 9, 310 10.3389/fcimb.2019.00310. 31555607 PMC6727670

[ref13] Lonergan Z. R.; Palmer L. D.; Skaar E. P. Histidine Utilization Is a Critical Determinant of *Acinetobacter* Pathogenesis. Infect. Immun. 2020, 88, e00118–e00120. 10.1128/IAI.00118-20. 32341119 PMC7309604

[ref14] Conde-Pérez K.; Vázquez-Ucha J. C.; Álvarez-Fraga L.; Ageitos L.; Rumbo-Feal S.; Martínez-Guitián M.; Trigo-Tasende N.; Rodríguez J.; Bou G.; Jiménez C.; et al. In-Depth Analysis of the Role of the Acinetobactin Cluster in the Virulence of *Acinetobacter baumannii*. Front. Microbiol. 2021, 12, 752070 10.3389/fmicb.2021.752070. 34675911 PMC8524058

[ref15] Read B. J.; Fisher G.; Wissett O. L. R.; Machado T. F. G.; Nicholson J.; Mitchell J. B. O.; da Silva R. G. Allosteric Inhibition of *Acinetobacter baumannii* ATP Phosphoribosyltransferase by Protein:Dipeptide and Protein:Protein Interactions. ACS Infect. Dis. 2022, 8, 197–209. 10.1021/acsinfecdis.1c00539. 34928596

[ref16] Gerhart J. C.; Pardee A. B. The Enzymology Of Control By Feedback Inhibition. J. Biol. Chem. 1962, 237, 891–896. 10.1016/S0021-9258(18)60389-8. 13897943

[ref17] Tacconelli E.; Carrara E.; Savoldi A.; Harbarth S.; Mendelson M.; Monnet D. L.; Pulcini C.; Kahlmeter G.; Kluytmans J.; Carmeli Y.; et al. Discovery, Research, And Development Of New Antibiotics: The WHO Priority List Of Antibiotic-Resistant Bacteria And Tuberculosis. Lancet Infect. Dis. 2018, 18, 318–327. 10.1016/S1473-3099(17)30753-3. 29276051

[ref18] Ayoub Moubareck C.; Hammoudi Halat D. Insights into *Acinetobacter baumannii*: A Review of Microbiological, Virulence, and Resistance Traits in a Threatening Nosocomial Pathogen. Antibiotics 2020, 9, 119 10.3390/antibiotics9030119. 32178356 PMC7148516

[ref19] Holdgate G. A.; Meek T. D.; Grimley R. L. Mechanistic Enzymology In Drug Discovery: A Fresh Perspective. Nat. Rev. Drug Discov. 2018, 17, 115–132. 10.1038/nrd.2017.219. 29192286

[ref20] Stroek R.; Ge Y.; Talbot P. D.; Glok M. K.; Bernas K. E.; Thomson C. M.; Gould E. R.; Alphey M. S.; Liu H.; Florence G. J.; et al. Kinetics and Structure of a Cold-Adapted Hetero-Octameric ATP Phosphoribosyltransferase. Biochemistry 2017, 56, 793–803. 10.1021/acs.biochem.6b01138. 28092443

[ref21] Alphey M. S.; Fisher G.; Ge Y.; Gould E. R.; Machado T. G.; Liu H.; Florence G. J.; Naismith J. H.; da Silva R. G. Catalytic And Anticatalytic Snapshots Of A Short-Form ATP Phosphoribosyltransferase. ACS Catal. 2018, 8, 5601–5610. 10.1021/acscatal.8b00867.

[ref22] Gibson D. G. Synthesis Of DNA Fragments In Yeast By One-Step Assembly Of Overlapping Oligonucleotides. Nucleic Acids Res. 2009, 37, 6984–6990. 10.1093/nar/gkp687. 19745056 PMC2777417

[ref23] Smith D. W.; Ames B. N. Phosphoribosyladenosine Monophosphate, An Intermediate In Histidine Biosynthesis. J. Biol. Chem. 1965, 240, 3056–3063. 10.1016/S0021-9258(18)97286-8. 14342333

[ref24] Salomaa P.; Schaleger L. L.; Long F. A. Solvent Deuterium Isotope Effects on Acid-Base Equilibria. J. Am. Chem. Soc. 1964, 86, 1–7. 10.1021/ja01055a001.

[ref25] Webb M. R. A Continuous Spectrophotometric Assay For Inorganic Phosphate And For Measuring Phosphate Release Kinetics In Biological Systems. Proc. Natl. Acad. Sci. U. S. A. 1992, 89, 4884–4887. 10.1073/pnas.89.11.4884. 1534409 PMC49192

[ref26] Cleland W. W. Partition Analysis And The Concept Of Net Rate Constants As Tools In Enzyme Kinetics. Biochemistry 1975, 14, 3220–3224. 10.1021/bi00685a029. 1148201

[ref27] Anderson K. S. Fundamental Mechanisms Of Substrate Channeling. Methods Enzymol. 1999, 308, 111–145. 10.1016/S0076-6879(99)08008-8. 10507003

[ref28] Pareek V.; Sha Z.; He J.; Wingreen N. S.; Benkovic S. J. Metabolic channeling: predictions, deductions, and evidence. Mol. Cell 2021, 81, 3775–3785. 10.1016/j.molcel.2021.08.030. 34547238 PMC8485759

[ref29] Gadda G.; Sobrado P. Kinetic Solvent Viscosity Effects as Probes for Studying the Mechanisms of Enzyme Action. Biochemistry 2018, 57, 3445–3453. 10.1021/acs.biochem.8b00232. 29874467

[ref30] Fisher G.; Thomson C. M.; Stroek R.; Czekster C. M.; Hirschi J. S.; da Silva R. G. Allosteric Activation Shifts the Rate-Limiting Step in a Short-Form ATP Phosphoribosyltransferase. Biochemistry 2018, 57, 4357–4367. 10.1021/acs.biochem.8b00559. 29940105 PMC6128619

[ref31] Schowen K. B.; Schowen R. L. Solvent Isotope Effects Of Enzyme Systems. Methods Enzymol. 1982, 87, 551–606. 10.1016/S0076-6879(82)87031-6. 6294457

[ref32] Fernandez P. L.; Murkin A. S. Inverse Solvent Isotope Effects in Enzyme-Catalyzed Reactions. Molecules 2020, 25, 1933 10.3390/molecules25081933. 32326332 PMC7221790

[ref33] Smith D. W.; Ames B. N. Intermediates In The Early Steps Of Histidine Biosynthesis. J. Biol. Chem. 1964, 239, 1848–1855. 10.1016/S0021-9258(18)91271-8. 14213364

[ref34] Konsowitz L. M.; Cooperman B. S. Solvent Isotope Effect In Inorganic Pyrophosphatase-Catalyzed Hydrolysis Of Inorganic Pyrophosphate. J. Am. Chem. Soc. 1976, 98, 1993–1995. 10.1021/ja00423a072. 3542

[ref35] Karsten W. E.; Lai C.-J.; Cook P. F. Inverse Solvent Isotope Effects In The NAD-Malic Enzyme Reaction Are The Result Of The Viscosity Difference Between D_2_O And H_2_O: Implications For Solvent Isotope Effect Studies. J. Am. Chem. Soc. 1995, 117, 5914–5918. 10.1021/ja00127a002.

[ref36] Merkler D. J.; Schramm V. L. Catalytic Mechanism Of Yeast Adenosine 5’-Monophosphate Deaminase. Zinc Content, Substrate Specificity, pH Studies, And Solvent Isotope Effects. Biochemistry 1993, 32, 5792–5799. 10.1021/bi00073a011. 8504099

[ref37] Pedreno S.; Pisco J. P.; Larrouy-Maumus G.; Kelly G.; de Carvalho L. P. Mechanism Of Feedback Allosteric Inhibition Of ATP Phosphoribosyltransferase. Biochemistry 2012, 51, 8027–8038. 10.1021/bi300808b. 22989207 PMC3466779

[ref38] Johnson K. A. 1 Transient-State Kinetic Analysis of Enzyme Reaction Pathways. In The Enzymes, Sigman D. S., Ed.; Academic Press, 1992; Vol. 20 pp. 1–61.

[ref39] Arora Verasztó H.; Logotheti M.; Albrecht R.; Leitner A.; Zhu H.; Hartmann M. D. Architecture And Functional Dynamics Of The Pentafunctional AROM Complex. Nat. Chem. Biol. 2020, 16, 973–978. 10.1038/s41589-020-0587-9. 32632294

[ref40] Pareek V.; Tian H.; Winograd N.; Benkovic S. J. Metabolomics And Mass Spectrometry Imaging Reveal Channeled De Novo Purine Synthesis In Cells. Science 2020, 368, 283–290. 10.1126/science.aaz6465. 32299949 PMC7494208

[ref41] Fani R.; Brilli M.; Fondi M.; Lió P. The Role Of Gene Fusions In The Evolution Of Metabolic Pathways: The Histidine Biosynthesis Case. BMC Evol. Biol. 2007, 7, S4 10.1186/1471-2148-7-S2-S4. PMC1963479 17767732

[ref42] Hidalgo-Zarco F.; Camacho A. G.; Bernier-Villamor V.; Nord J.; Ruiz-Pérez L. M.; González-Pacanowska D. Kinetic Properties And Inhibition Of The Dimeric dUTPase- dUDPase From *Leishmania major*. Protein Sci. 2001, 10, 1426–1433. 10.1110/ps.48801. 11420444 PMC2374113

[ref43] Moroz O. V.; Murzin A. G.; Makarova K. S.; Koonin E. V.; Wilson K. S.; Galperin M. Y. Dimeric dUTPases, HisE, And MazG Belong To A New Superfamily Of All-Alpha NTP Pyrophosphohydrolases With Potential ″House-Cleaning″ Functions. J. Mol. Biol. 2005, 347, 243–255. 10.1016/j.jmb.2005.01.030. 15740738

[ref44] García-Nafría J.; Harkiolaki M.; Persson R.; Fogg M. J.; Wilson K. S. The Structure Of *Bacillus subtilis* Spβ Prophage dUTPase And Its Complexes With Two Nucleotides. Acta Crystallogr. D Biol. Crystallogr. 2011, 67, 167–175. 10.1107/S0907444911003234. 21358047

[ref45] Dauter Z.; Persson R.; Rosengren A. M.; Nyman P. O.; Wilson K. S.; Cedergren-Zeppezauer E. S. Crystal Structure Of dUTPase From Equine Infectious Anaemia Virus; Active Site Metal Binding In A Substrate Analogue Complex. J. Mol. Biol. 1999, 285, 655–673. 10.1006/jmbi.1998.2332. 9878436

