# Supplementary material for: Catalytic Cycle of the Bifunctional Enzyme Phosphoribosyl-ATP Pyrophosphohydrolase/Phosphoribosyl-AMP Cyclohydrolase
Source: ACS Catal. 2023 May 23;13(11):7669–79. doi: 10.1021/acscatal.3c01111 (PMC10242683; doi:10.1021/acscatal.3c01111)
Supplement: Supplementary file 1 — cs3c01111_si_001.pdf [file cs3c01111_si_001.pdf]

Supporting Information

The catalytic cycle of the bifunctional enzyme phosphoribosyl-ATP pyrophosphohydrolase/phosphoribosyl-AMP cyclohydrolase

Gemma Fisher, Ennio Pečaver, Benjamin J. Read, Susannah K. Leese, Erin Laing, Alison L. Dickson, Clarissa M. Czekster, and Rafael G. da Silva*

School of Biology, Biomedical Sciences Research Complex, University of St Andrews, St Andrews, Fife KY16 9ST, United Kingdom

*To whom correspondence may be addressed: rgds@st-andrews.ac.uk, phone: +44 (0)1334 463496

RESULTS AND DISCUSSION

Purification and biophysical characterisation of *Ab*HisIE and *Ab*HisE^{domain}. *Ab*HisIE was purified to homogeneity as estimated by Coomassie Blue-stained SDS-PAGE (Figure S1A), and bands highlighted in the gel were confirmed to be monomeric and dimeric forms of *Ab*HisIE by trypsin digestion followed by LC-MS/MS analysis of the tryptic peptides (Figure S1B). DSF-based thermal denaturation of *Ab*HisIE showed a complex pattern where different portions of the protein denature at different temperatures, which was unchanged in the presence of PRATP (Figure S2), suggesting no thermal stabilisation by substrate/product. The C-terminal domains of *S. flexneri* and *M. truncatula* are composed strictly of α-helices, while their N-terminal domains have a mixture of α-helices and β-sheets,[1,2] and *Ab*HisIE shares 41% and 32% amino acid sequence identity with those proteins, respectively (Figure S3). An AlphaFold[3] model of *Ab*HisIE (Figure S4A) was used to help the design of *Ab*HisE^{domain}, whose amino acid sequence and AlphaFold model are shown in Figure S4B.

*Ab*HisE^{domain} was purified to homogeneity as estimated by Coomassie Blue-stained SDS-PAGE (Figure S5A), and ESI-MS analysis revealed a mass of 12945 (Figure S5B), 17 mass units higher than the theoretical value based on the amino acid sequence, possibly resulting from a methionine residue being oxidised. DSF-based thermal denaturation of *Ab*HisIE fitted to a Boltzmann equation[3] yielded a melting temperature of 62.3 ± 0.1 °C (Figure S6).

Enzymatic synthesis of PRATP, PRADP, and PRAMP. PRATP, PRADP, and PRAMP were successfully synthesized biocatalylically and purified by anion-exchange chromatography (Figure S7). EIS-MS analysis of the purified compounds showed masses matching the corresponding predicted values (Figure S7).

***Ab*HisIE-catalysed formation of ProFAR from PRATP.** When 40 μM PRATP is incubated with *Ab*HisIE at 25 °C, the reaction reaches completion after ~600 s, and ~40 μM

ProFAR is produced (Figure S8A), in agreement with the expected irreversibility of the overall reaction. Orthogonal confirmation of ProFAR formation from PRATP was obtained by LC-MS analysis of the reaction, where a species with m/z matching the predicted $[M-H]^-$ for ProFAR was detected (Figure S8B). Addition of $ZnCl_2$ led to inhibition of the reaction (Figure S9A), but $MgCl_2$ was required for activity (Figure S9B), as previously observed for HisI.[4] The initial rate increased linearly with increasing *Ab*HisIE concentration (Figure S10).

***Ab*HisIE- and *Ab*HisE^{domain}-catalysed formation of PP_i from PRATP.** *Ab*HisIE-catalysed PP_i formation by the pyrophosphohydrolase moiety of *Ab*HisIE was assessed independently of the cyclohydrolase activity with the EnzChek Pyrophosphate Assay kit (Scheme S1). A series of controls were performed to demonstrate the background signal due to possible trace amount of phosphate in the *Ab*HisIE and/or PRATP preparations is negligible and the assay reports on the rate of *Ab*HisIE. No PP_i was detected in the absence of either PRATP or PPase, and only ~200 nM PPi was detected in the absence of *Ab*HisIE, likely originating from the PRATP preparation. When all components were added, initial rate of PP_i formation could be measured (Figure S11), and it increased linearly with increasing *Ab*HisIE concentration (Figure S12).

The synthesis of PRAMP indicated that *Ab*HisE^{domain} was catalytically active (Figure S7). To confirm the catalytic activity of *Ab*HisE^{domain} in a systematic manner, PP_i formation was assayed in a similar way as carried out for *Ab*HisIE, and controls lacking either *Ab*HisE^{domain}, PRATP, or PPase failed to produce PP_i. Only when all components were added was PP_i formation detected, with initial rates that tracked with *Ab*HisE^{domain} concentration (Figure S13).

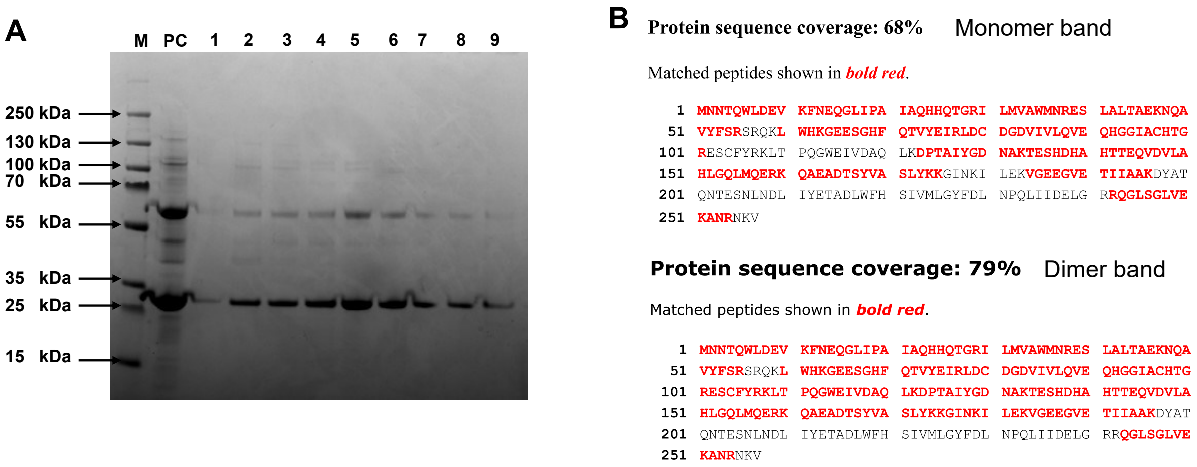

Figure S1. Purification of *Ab*HisIE. (A) Coomassie Blue-stained SDS-PAGE of purified *Ab*HisIE after size-exclusion chromatography. Lane M denotes PageRuler Plus Prestained Protein Ladder, the molecular weight marker. Lane PC, the sample that was loaded onto the column. Lanes 1 – 9, the eluate from the column. (B) Trypsin digestion of each band and subsequent peptide mapping by LC-MS/MS analysis identified both bands at ~30 kDa and at ~60 kDa as *Ab*HisIE.

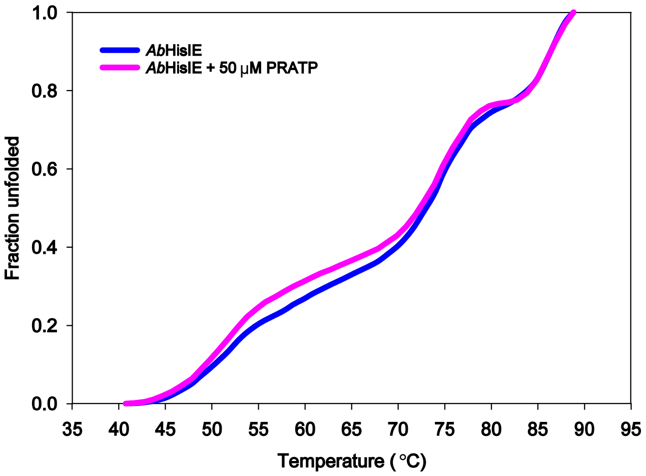

Figure S2. DSF-based thermal denaturation of *Ab*HisIE in the absence and presence of PRATP.

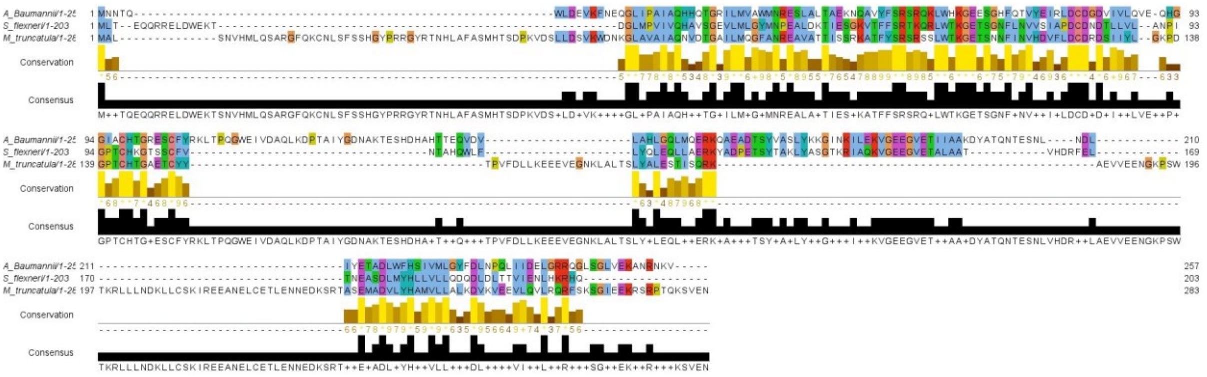

Figure S3. Amino acid sequence alignment among HisIE from *A. baumannii*, *S. flexneri*, and *M. truncatula*. Alignment performed in Clustal 2.1 and image generated in Jalview.

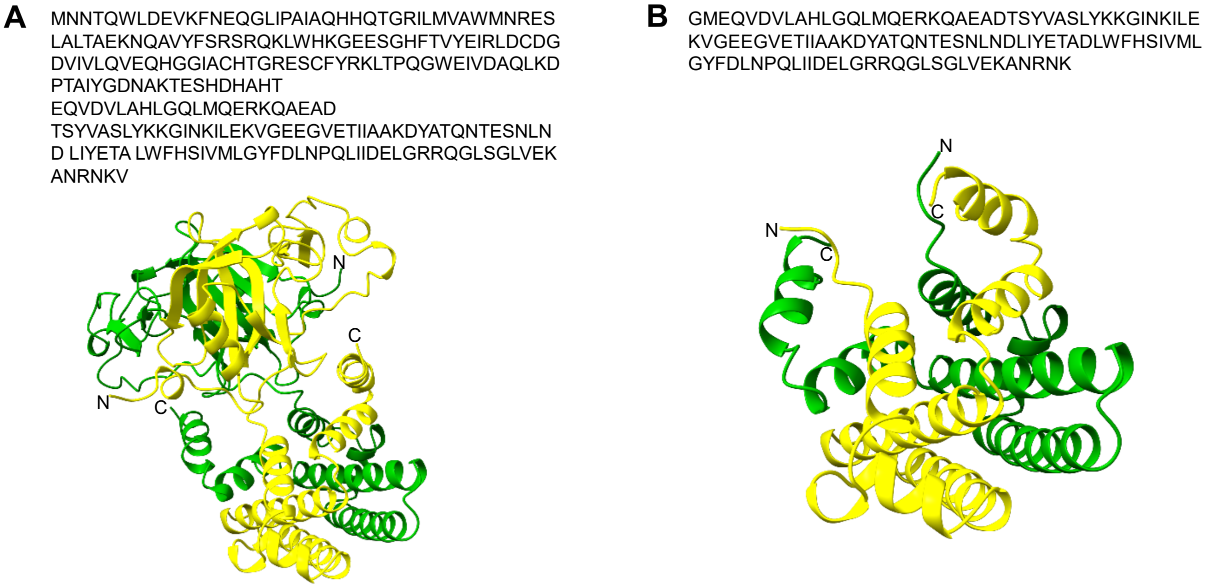

Figure S4. Three-dimensional models. (A) Amino acid sequence (top) and ribbon diagram of the AlphaFold 3-D model of *Ab*HisIE dimer (bottom). (B) Amino acid sequence (top) and ribbon diagram of the AlphaFold 3-D model of the designed *Ab*HisE^{domain} dimer (bottom). The N-terminal glycine residue in the *Ab*HisE^{domain} sequence is left upon TEVP cleavage. In all cases, individual monomers are depicted in yellow and green, and N and C indicate the N- and C-termini of the polypeptide chains.

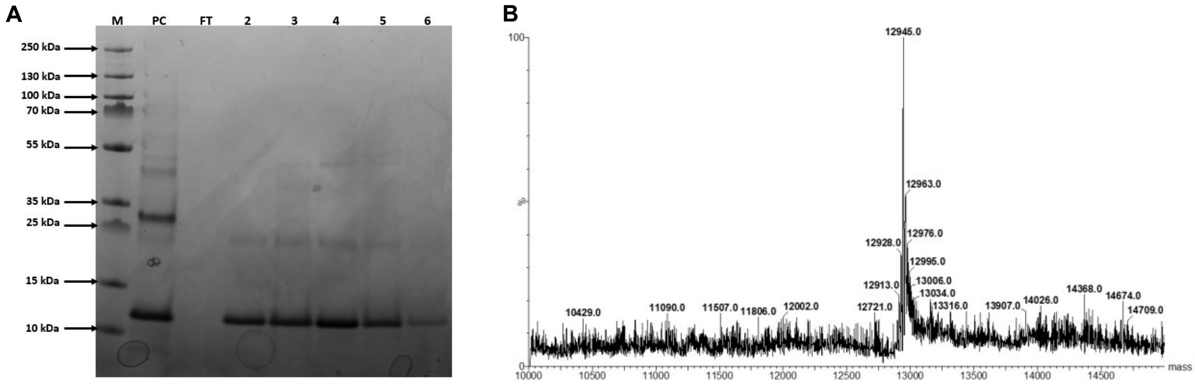

Figure S5. Purification of *Ab*HisE^{domain}. (A) Coomassie Blue-stained SDS-polyacrylamide gel of purified *Ab*HisE^{domain} after size-exclusion chromatography. Lane M denotes PageRuler Plus Prestained Protein Ladder, the molecular weight marker. Lane PC, the sample that was loaded onto the column. Lane FT is the flow through. Lanes 1 – 6, the eluate from the column. (B) ESI-MS analysis of purified *Ab*HisE^{domain}.

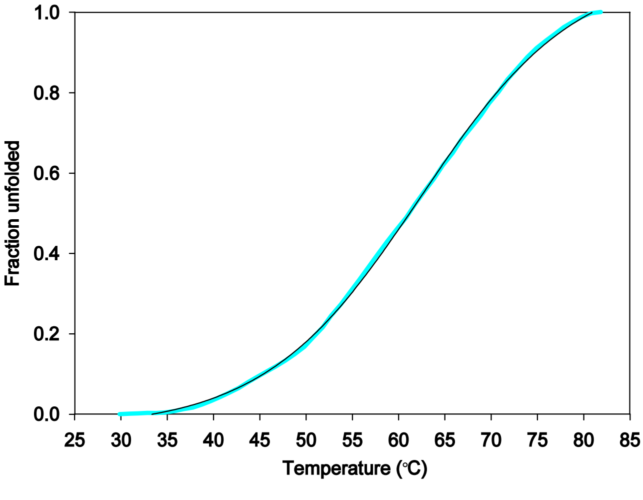

Figure S6. DSF-based thermal denaturation of *Ab*HisE^{domain}. The black line is the best fit to a Boltzmann equation.

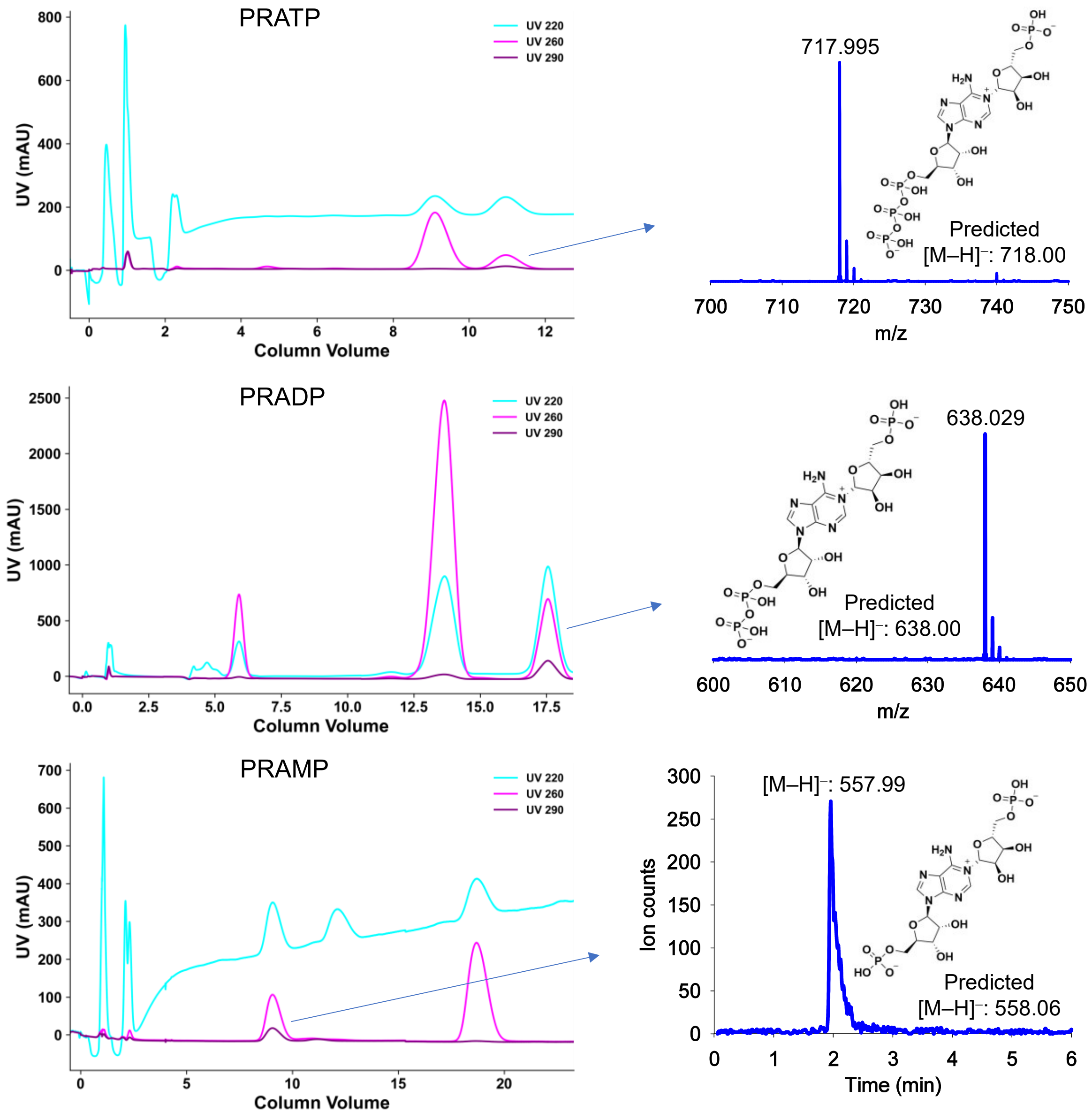

Figure S7. Representative chromatograms (left) and ESI-MS analysis (right) of PRATP, PRADP, and PRAMP. In insets on the right depict a possible ionisation form of each compound that could produce the corresponding molecular ion detected.

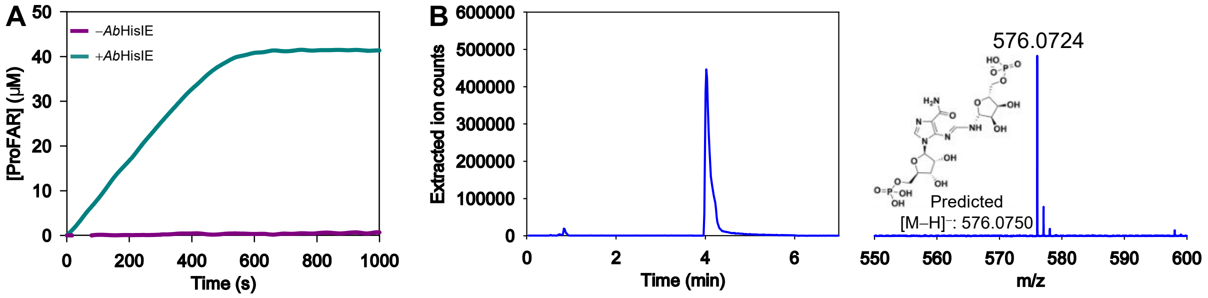

Figure S8. Analysis of the *Ab*HisIE-catalysed formation of ProFAR from PRATP. (A) Time course of ProFAR formation from 40 μM PRATP in the presence and absence of *Ab*HisIE. (B) Elution profile of extracted ions of mass 576.07 ± 0.05 (left), and high-resolution m/z spectrum of the peak eluted at ~4.02 min (right). The inset depicts the possible single-charge state of ProFAR detected in negative mode whose predicted m/z matches the detected m/z to 4.5 ppm.

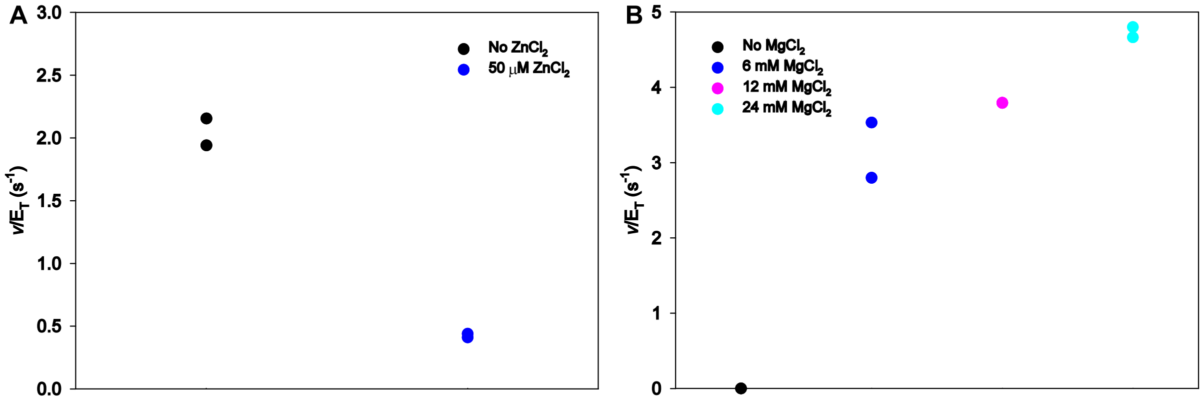

Figure S9. Effect of divalent metals on *Ab*HisIE activity. (A) *Ab*HisIE activity upon addition of $ZnCl_2$ to assay mixture (13.5 μM PRATP; 12 mM $MgCl_2$). (B) *Ab*HisIE activity in the presence and absence of $MgCl_2$ in the assay mixture (37 μM PRATP). All data points are shown.

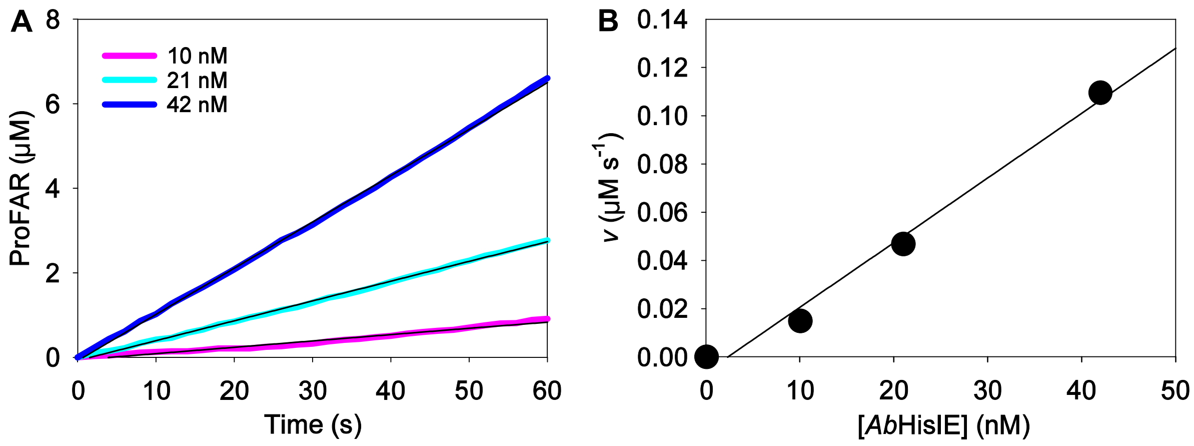

Figure S10. Initial rate dependence on *Ab*HisIE concentration. (A) ProFAR formation time course. Thick lines are mean traces from two independent measurements; thin black lines are linear regressions of the data. (B) Initial rate (from linear regression of the data in A) dependence on *Ab*HisIE concentration. Each data point is rate ± fitting error. The line is a linear regression of the data.

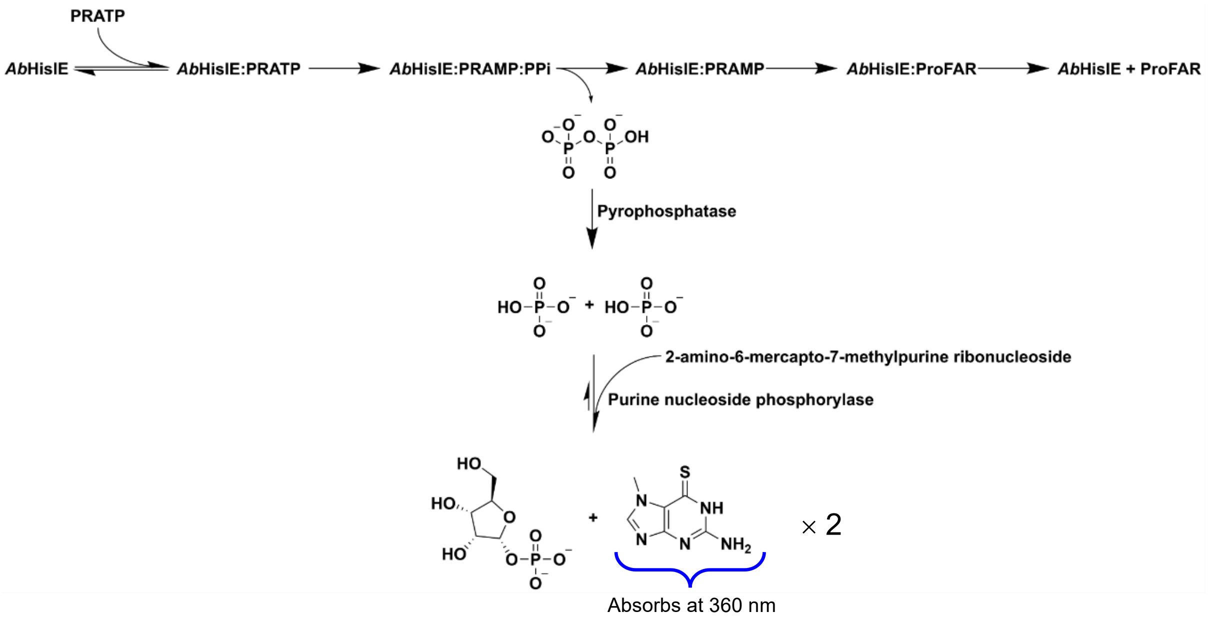

Scheme S1. Continuous assay of PP_i formation by the pyrophosphohydrolase moiety of *Ab*HisIE via the EnzChek Pyrophosphate Assay kit.

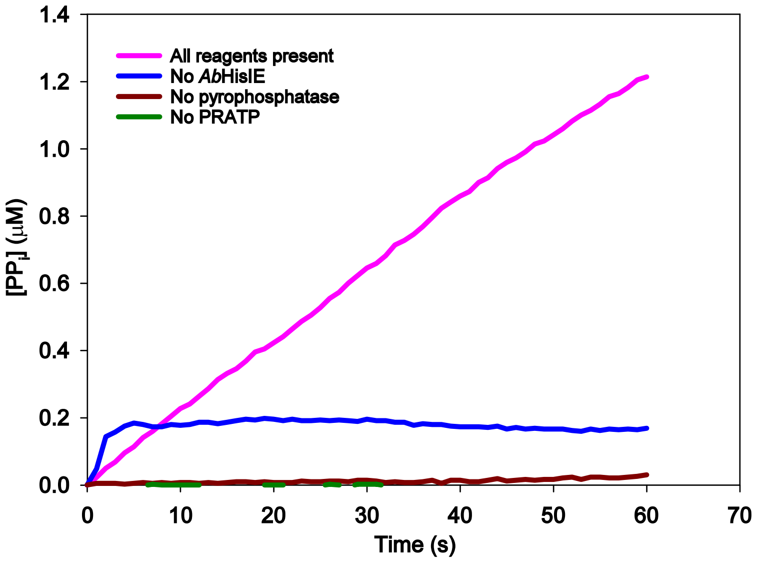

Figure S11. Time course of PP_i formation based on the EnzChek Pyrophosphate Assay kit. Traces are averages of two independent measurements. For the reaction mixture with all reagents present, *Ab*HisIE was added last to the cuvette, 3 min after all other reagents, to allow the background signal from PRATP to plateau.

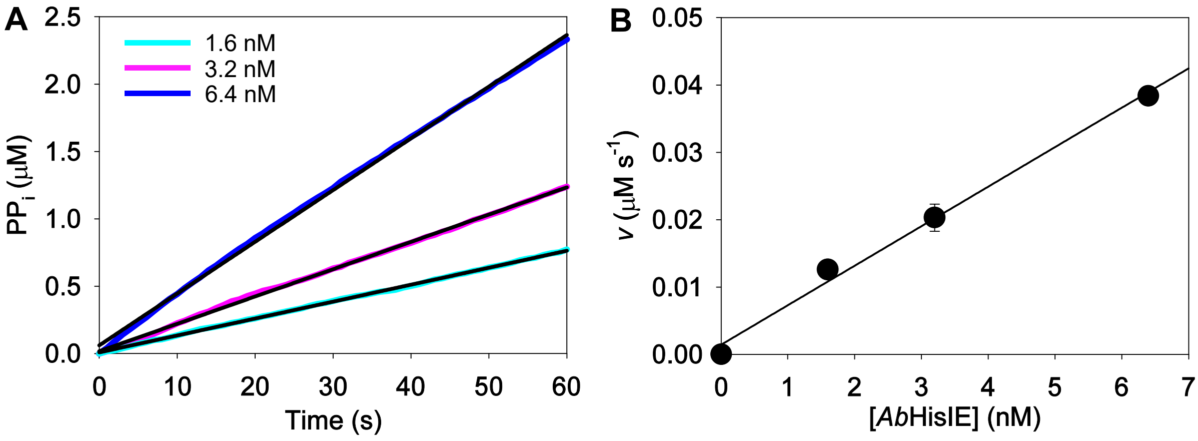

Figure S12. Initial rate dependence on *Ab*HisIE concentration. (A) PP_i formation time course. Thick lines are mean traces from two independent measurements; thin black lines are linear regressions of the data. (B) Initial rate (from linear regression of the data in A) dependence on *Ab*HisIE concentration. Each data point is rate ± fitting error. The line is a linear regression of the data.

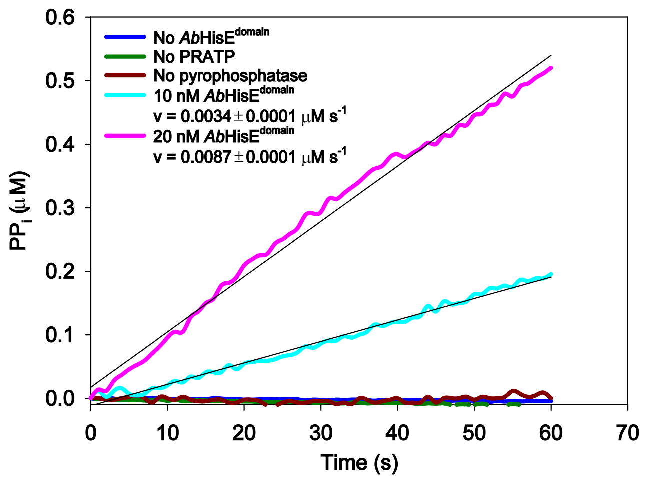

Figure S13. Time course of PP_i formation based on the EnzChek Pyrophosphate Assay kit. Traces are averages of two independent measurements. For the reaction mixtures with all reagents present, *Ab*HisE^{domain} was added last to the cuvette, 3 min after all other reagents. Thin black lines are linear regression of the traces with all reagents present, from which initial rates (*v*) were calculated.

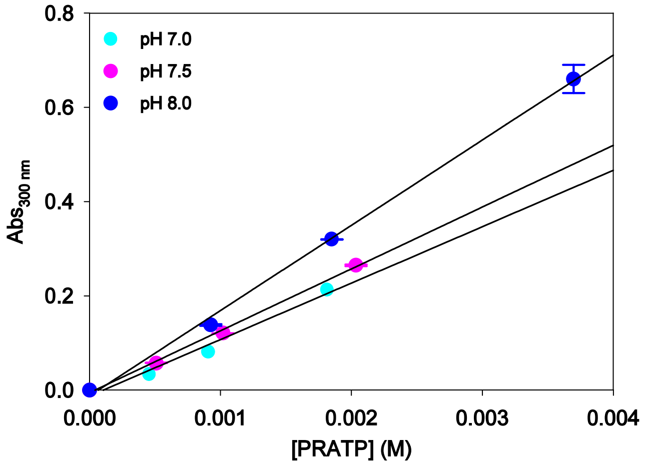

Figure S14. Determination of the molar extinction coefficient of PRATP at 300 nm at different pH values. Data points are mean ± standard deviation from three independent measurements. Lines represent linear regressions of the data. The path length for absorbance measurements was 0.1 cm.

Table S1. Solvent viscosity effects on *Ab*HisIE steady-state kinetic parameters for ProFAR formation.

Parameter	0% glycerol	9% glycerol	18% glycerol
k_{cat}^{ProFAR} (s^{-1})	5.6 ± 0.3	6.1 ± 0.2	5.7 ± 0.4
K_M (μM)	4.0 ± 0.7	4.5 ± 0.5	4 ± 1
k_{cat}^{ProFAR}/K_M (M^{-1} s^{-1})	(1.4 ± 0.3) × 10^6	(1.4 ± 0.2) × 10^6	(1.4 ± 0.4) × 10^6

Table S2. Molar extinction coefficients at 300 nm (ε_{300}) for PRATP and $\Delta\varepsilon_{300}$ for ProFAR.

pH	$\varepsilon_{300}^{PRATP}$ (M^{-1} cm^{-1})[a]	$\Delta\varepsilon_{300}^{ProFAR}$ (M^{-1} cm^{-1})[b]	Rounded $\Delta\varepsilon_{300}^{ProFAR}$ (M^{-1} cm^{-1})
**7.0**	1200 ± 100	6800	6800
**7.5**	1310 ± 50	6690	6700
**8.0**	1810 ± 50	6190	6200

[a]Slope ± fitting error from the linear regressions in Figure S12.

[b]The $\Delta\varepsilon_{300}$ is calculated by subtracting each $\varepsilon_{300\,nm}$ value from 8000 M^{-1} cm^{-1}.

Derivation of equations 5 – 9

For Scheme 2

Net rate constants:

$$k'_{11} = k_{11}\ ;\ k'_9 = k_9\ ;\ k'_7 = \frac{k_7 PRAMP k_9}{k_8 + k_9}\ ;\ k'_5 = k_5\ ;\ k'_3 = k_3\ ;\ k'_1 = \frac{k_1 PRATP k_3}{k_2 + k_3}$$

Proportion of free enzyme:

$$\frac{E}{E_T} = \frac{\frac{1}{k'_1}}{\frac{1}{k'_1} + \frac{1}{k'_3} + \frac{1}{k'_5} + \frac{1}{k'_7} + \frac{1}{k'_9} + \frac{1}{k'_{11}}}$$

Initial rate:

$v = k'_1 E$

$$v = k'_1 \left(\frac{\frac{1}{k'_1}}{\frac{1}{k'_1}+\frac{1}{k'_3}+\frac{1}{k'_5}+\frac{1}{k'_7}+\frac{1}{k'_9}+\frac{1}{k'_{11}}} \right) E_T$$

$$\frac{v}{E_T} = \frac{1}{\frac{1}{k'_1}+\frac{1}{k'_3}+\frac{1}{k'_5}+\frac{1}{k'_7}+\frac{1}{k'_9}+\frac{1}{k'_{11}}}$$

Detecting PP_i when PRATP levels are saturating (the cyclohydrolase reaction is irrelevant, and only net rate constants not containing PRATP remain):

$$k_{cat} = \frac{1}{\frac{1}{k'_3}+\frac{1}{k'_5}}$$

Detecting PP_i when PRATP levels tend to zero (the cyclohydrolase reaction is irrelevant, and only net rate constants containing PRATP remain):

$$\frac{k_{cat}}{K_M} = \frac{1}{\frac{1}{k'_1}}$$

Substituting the net rate constant definitions for the respective net rate constants and carrying out some simple algebraic operations:

$$k_{cat}^{PP_i} = \frac{k_3 k_5}{k_3+k_5} \qquad \text{eq 5}$$

$$\frac{k_{cat}^{PP_i}}{K_M} = \frac{k_1 k_3}{k_2+k_3} \qquad \text{eq 6}$$

Detecting ProFAR when PRATP levels are saturating (only net rate constants not containing PRATP remain):

$$k_{cat} = \frac{1}{\frac{1}{k'_3}+\frac{1}{k'_5}+\frac{1}{k'_9}+\frac{1}{k'_{11}}}$$

Detecting ProFAR when PRATP levels tend to zero (only net rate constants containing PRATP remain):

$$\frac{k_{cat}}{K_M} = \frac{1}{\frac{1}{k'_1}+\frac{1}{k'_7}}$$

Substituting the net rate constants definition for the respective net rate constants and carrying out some simple algebraic operations:

$$k_{cat}^{ProFAR} = \frac{k_3k_5k_9k_{11}}{k_3k_5k_9+k_{11}(k_3k_5+k_3k_9+k_5k_9)} \quad \text{eq 7}$$

$$\frac{k_{cat}^{ProFAR}}{K_M} = \frac{k_1k_3k_7k_9}{k_1k_3k_8+k_9(k_1k_3+k_2k_7+k_3k_7)} \quad \text{eq 8}$$

For Scheme 3

Net rate constants:

$$k'_{13} = k_{13}\ ;\ k'_{11} = k_{11}\ ;\ k'_9 = k_9\ ;\ k'_7 = \frac{k_7PRAMPk_9}{k_8+k_9}\ ;\ k'_5 = k_5\ ;\ k'_3 = k_3\ ;\ k'_1 = \frac{k_1PRATPk_3}{k_2+k_3}$$

Proportion of free enzyme:

$$\frac{E}{E_T} = \frac{\frac{1}{k'_1}}{\frac{1}{k'_1}+\frac{1}{k'_3}+\frac{1}{k'_5}+\frac{1}{k'_7}+\frac{1}{k'_9}+\frac{1}{k'_{11}}+\frac{1}{k'_{13}}}$$

Initial rate:

$$v = k'_1E$$

$$v = k'_1\left(\frac{\frac{1}{k'_1}}{\frac{1}{k'_1}+\frac{1}{k'_3}+\frac{1}{k'_5}+\frac{1}{k'_7}+\frac{1}{k'_9}+\frac{1}{k'_{11}}+\frac{1}{k'_{13}}}\right)E_T$$

$$\frac{v}{E_T} = \frac{1}{\frac{1}{k'_1}+\frac{1}{k'_3}+\frac{1}{k'_5}+\frac{1}{k'_7}+\frac{1}{k'_9}+\frac{1}{k'_{11}}+\frac{1}{k'_{13}}}$$

Detecting ProFAR when PRATP levels are saturating (only net rate constants not containing PRATP remain):

$$k_{cat} = \frac{1}{\frac{1}{k'_3}+\frac{1}{k'_5}+\frac{1}{k'_9}+\frac{1}{k'_{11}}+\frac{1}{k'_{13}}}$$

Substituting the net rate constant definitions for the respective net rate constants and carrying out some simple algebraic operations:

$$k_{cat}^{ProFAR} = \frac{k_3k_5k_9k_{11}k_{13}}{k_3k_5k_9k_{11}+k_{13}(k_3k_5k_{11}+k_3k_9k_{11}+k_5k_9k_{11}+ k_3k_5k_9)} \quad \text{eq 9}$$

REFERENCES

(1) Wang, Y.; Zhang, F.; Nie, Y.; Shang, G.; Zhang, H. Structural Analysis Of *Shigella Flexneri* Bi-Functional Enzyme HisIE In Histidine Biosynthesis. *Biochem. Biophys. Res. Commun.* **2019**, *516*, 540-545.

(2) Witek, W.; Sliwiak, J.; Ruszkowski, M. Structural And Mechanistic Insights Into The Bifunctional HISN2 Enzyme Catalyzing The Second And Third Steps Of Histidine Biosynthesis In Plants. *Sci. Rep.* **2021**, *11*, 9647.

(3) Jumper, J.; Evans, R.; Pritzel, A.; Green, T.; Figurnov, M.; Ronneberger, O.; Tunyasuvunakool, K.; Bates, R.; Žídek, A.; Potapenko, A.; et al. Highly Accurate Protein Structure Prediction With Alphafold. *Nature* **2021**, *596*, 583-589.

(4) D'Ordine, R. L.; Klem, T. J.; Davisson, V. J. N1-(5'-Phosphoribosyl)Adenosine-5'-Monophosphate Cyclohydrolase: Purification And Characterization Of A Unique Metalloenzyme. *Biochemistry* **1999**, *38*, 1537-1546.
